# Broad specificity of immune helminth scFv library to identify monoclonal antibodies targeting *Strongyloides*

**DOI:** 10.1038/s41598-021-82125-3

**Published:** 2021-01-28

**Authors:** Anizah Rahumatullah, Dinesh Balachandra, Rahmah Noordin, Zamrina Baharudeen, Yee Ying Lim, Yee Siew Choong, Theam Soon Lim

**Affiliations:** Institute for Research in Molecular Medicine (INFORMM), Universiti Sains Malaysia, 11800 Minden, Penang Malaysia

**Keywords:** Infectious diseases, Immunological techniques

## Abstract

Antibodies have different chemical properties capable of targeting a diverse nature of antigens. Traditionally, immune antibody libraries are perceived to be disease-specific with a skewed repertoire. The complexity during the generation of a combinatorial antibody library allows for a skewed but diverse repertoire to be generated. *Strongyloides stercoralis* is a parasite that causes strongyloidiasis, a potentially life-threatening disease with a complex diagnosis that impedes effective control and treatment of the disease. This study describes the isolation of monoclonal antibodies against *S. stercoralis* NIE recombinant protein using an immune antibody phage display library derived from lymphatic filaria-infected individuals. The isolated antibody clones showed both lambda and kappa light chains gene usage, with diverse amino acid distributions. Structural analysis showed that electropositivity and the interface area could determine the binding affinity of the clones with NIE. The successful identification of *S. stercoralis* antibodies from the filarial immune library highlights the breadth of antibody gene diversification in an immune antibody library that can be applied for closely related infections.

## Introduction

In the past decade, phage display technology has become a common tool for the discovery of novel binders against various antigen targets^1^. The nature of the target antigen can range from proteins, haptens, peptides, enzymes, membrane fractions, liposomes, virus-like particles, cells, tissue sections, or entire tissues^2,3^. Phage display technology has been the preferred in vitro method for the production of monoclonal antibodies replacing the conventional hybridoma technology. Central to phage display technology is the construction of a phage library displaying a plethora of unique antibodies. An essential aspect of antibody libraries is the source of the antibody repertoire used for library preparation. Phage libraries can be divided into two main types, i.e., natural libraries that consist of antibody genes from immune and non-immune (naïve) donors, and synthetic libraries comprising antibody genes derived from chemical synthesis.

A naïve antibody library repertoire is derived from the IgM repertoire of donors in a healthy state. The advantage of this library is that it can be used for discovery of antibodies against a wide array of antigens. However, the drawback of naïve library-derived antibodies is a general lower affinity compared to antibodies from an immune source and a higher possibility of cross-reactions^4,5^. Nevertheless, this limitation may be circumvented by in vitro affinity maturation processes. Conversely, an immune library is derived from the IgG repertoire from an infected host. The antibody repertoire in immune libraries consists of B cells that have been exposed to a particular pathogen and have undergone affinity maturation processes^2^. Thus, the resulting antibodies have an increased affinity towards the target antigen, enabling isolation of high-affinity binders. The antibody repertoire from an immune source is generally of a lower diversity making immune libraries not as broadly applicable in terms of their antigen scope as compared to naïve libraries.

In the present study, we utilized a previously constructed Human AntibodY Disease ENhanced (HAYDEN)-Filariasis library which is an immune helminth phage display library^6^ to isolate monoclonal antibodies against *Strongyloides stercoralis* (*S. stercoralis*) NIE recombinant protein (rNIE). This parasite causes strongyloidiasis, a potentially life-threatening disease with a complex diagnosis that impedes effective control and treatment of the disease. The helminth library was generated using the blood of individuals infected with lymphatic filaria, specifically *Brugia malayi* (*B. malayi*). The samples were taken from apparently asymptomatic individuals with parasite larvae (microfilariae) in their blood. Parasites causing lymphatic filariasis (LF) and strongyloidiasis can be found in the blood and tissue of infected individuals. They are both helminths under the phylum of Nematoda (roundworm)^7^. Infection with helminth parasites are commonly associated with an elevation of IgE antibodies, eosinophilia, mucous mastocytosis, and goblet cells hyperplasia^8^. During helminth infection, T helper cell 2 (Th2) type response mediates protection, while B cells play vital roles in antibody secretion^9^, stimulation and control of Th2-type immune responses^10^. Following infection, antibodies such as IgG and IgM can act as potent mediators of protective immunity, and Th2-type responses trigger B cell class switching to IgE and IgG4^11^. The commonalities of immune responses of both parasites indicate the possibility of using the antibody library against *B. malayi* to isolate antibodies against *S. stercoralis*.

*Strongyloides stercoralis*, a human parasitic roundworm is estimated to infect approximately 370 million^12^ people globally, with a mortality rate of 16.7% among patients requiring hospitalization. Meanwhile, among immunocompromised patients, the fatality is 60–85%^11^. *S. stercoralis* mainly infects humans through the penetration of infective filariform larvae into the skin when there is contact with contaminated soil. This nematode’s autoinfection mechanism allows infection to be maintained life long as it is able to carry out and restart its life cycle within the human body. Immunosuppressed patients are most at risk of developing the life-threatening clinical symptoms of strongyloidiasis due to hyperinfection syndrome or dissemination^13^. Therefore, the treatment of *S. stercoralis* infections must completely eradicate the parasite from the body to avoid complications of hyperinfection and dissemination syndrome which may prove to be fatal. Strongyloidiasis is listed as a neglected tropical disease (NTD) by the World Health Organization (WHO) in 2004 and more often, the diagnostics of this disease is often overlooked as it requires the utility of complex diagnostic techniques and resources. The difficulty of diagnosing this disease is mainly due to the sporadic output of larvae and serological cross-reactivity to other parasitic infections.

In the present study, the protein used for biopanning was rNIE, a highly antigenic *S. stercoralis*-specific antigen used for serodiagnosis of strongyloidiasis. It was derived from an *S. stercoralis* L3 cDNA library^14^. Based on its high diagnostic specificity and sensitivity, rNIE has been commonly used for development of antibody detection assays for strongyloidiasis^15,16^.

The inherent ability of an immune library generated utilizing the antibody repertoire of a closely related infection, to present useful repertoire for antibody generation is not well-studied. The ability of the LF immune library to isolate *S. stercoralis*-specific antibodies demonstrates a broader coverage of immune libraries than currently understood, thus making it an even more valuable asset for antibody generation. This study highlights the immense genetic diversity of the antibody repertoire in an immune antibody library that yields antibodies against a related organism.

## Results

### Recombinant protein preparation and verification

rNIE was expressed and purified for biopanning. The protein was successfully expressed and verified using SDS PAGE and Western blot with an expected size of approximately 37 kDa (Fig. S1). The analysis showed the rNIE was produced with good yield and purity suitable for biopanning.

### Isolation of monoclonal antibodies

**Figure 1:**
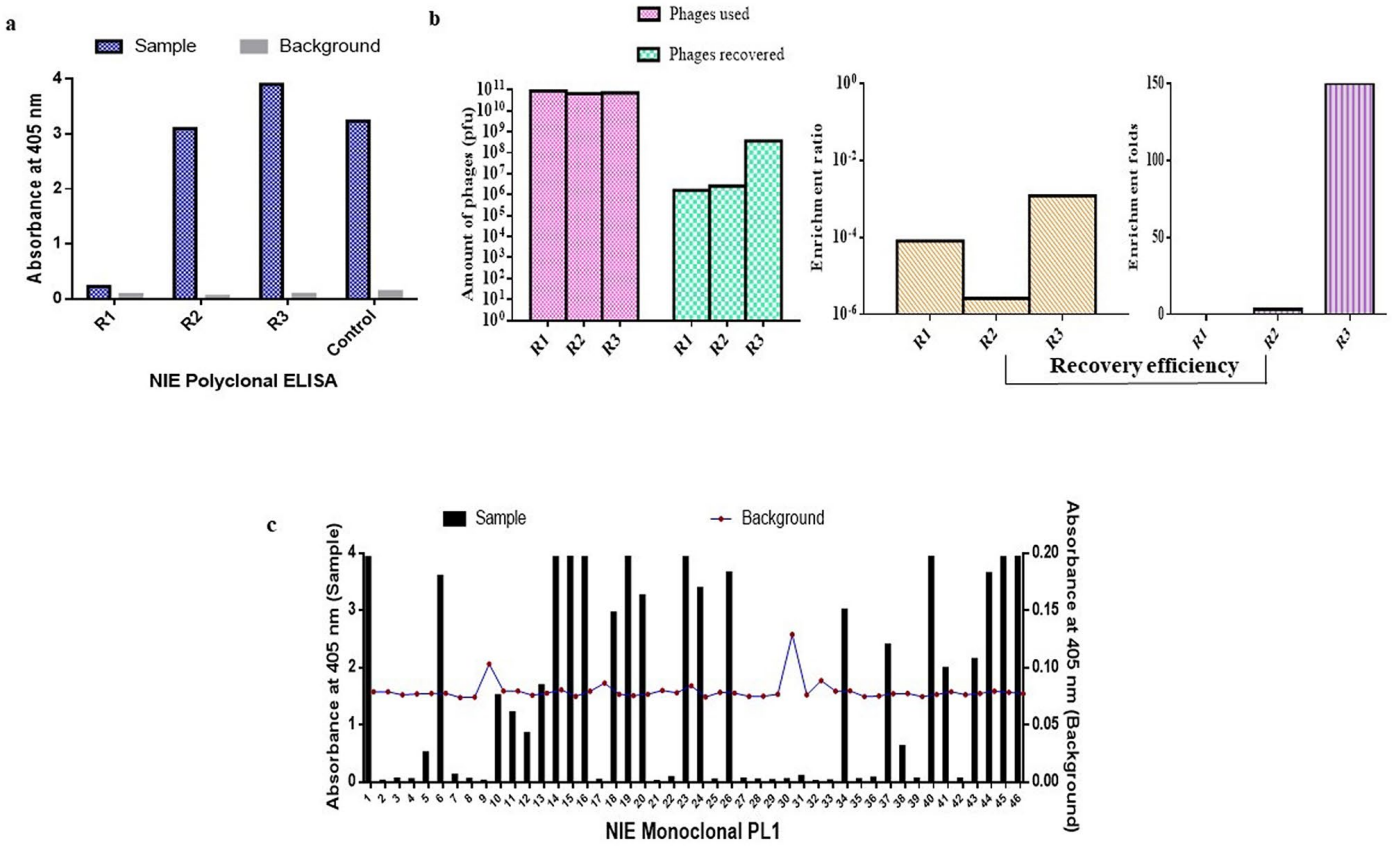
Isolation of rNIE specific monoclonal antibodies. (**a**) Polyclonal phage ELISA of rNIE antigen during the panning cycles. (**b**) Phage particles used and recovered in rounds 1 to 3 panning. (**c**) Representative of monoclonal phage ELISA analysis of scFv clones from helminth library against rNIE antigen. The positive control is a non-related protein that is reactive to the anti-M13-HRP antibodies and BSA was used as the negative control.

Monoclonal antibody clones against *Strongyloides* rNIE were isolated using previously described biopanning method^6^. The polyclonal ELISA results showed enrichment of rNIE-specific phage in consecutive rounds of the selection process and a significant increase in absorbance value approximately at 3.0 (Fig. 1a). Figure 1b shows the amount of phage used and recovered from each round of panning. The amount of phage particles from rounds 1 to 3 were 8.71 × 10^10^, 5.1 × 10^10^ and 1.21 × 10^10^, respectively. The amount of recovered phage particles in rounds 1 to 3 was 1.6 × 10^6^, 2.6 × 10^6^ and 3.6 × 10^8^ pfu, respectively. The enrichment ratios for rounds 1 to 3 were determined to be 8 × 10^–5^, 3 × 10^–6^ and 1.2 × 10^3^, respectively. This was translated to an enrichment ratio of 3.25 and 150 folds for rounds 2 and 3, respectively.

A total of 184 antibody clones were screened in the monoclonal ELISA (Fig. 1c) and this resulted in 104 positive binders with absorbance values ranging from 0.61 to 3.95. Positive clones were identified with a cut-off OD_405nm_ value above 0.5 after subtracting the background values with the majority of clones having OD_405nm_ readings above 2.5. This indicates that the helminth phage display library was capable of enriching rNIE- specific monoclonal antibodies.

### Antibody gene analysis

Sequencing results of the positive binders were analysed by IMGT/V-QUEST software to determine the identity of the scFv clones based on the human germline sequences available in the database. The sequence analysis showed that only 30 full-length antibody clones were obtained. The remaining 60 clones had only partial scFv sequences with either a complete heavy or light chain with complete complementarity-determining regions (CDR), while another 14 clones had mutations that resulted in frameshifts and truncations. These problematic clones were omitted from the gene pairing and antibody gene analysis.

**Figure 2:**
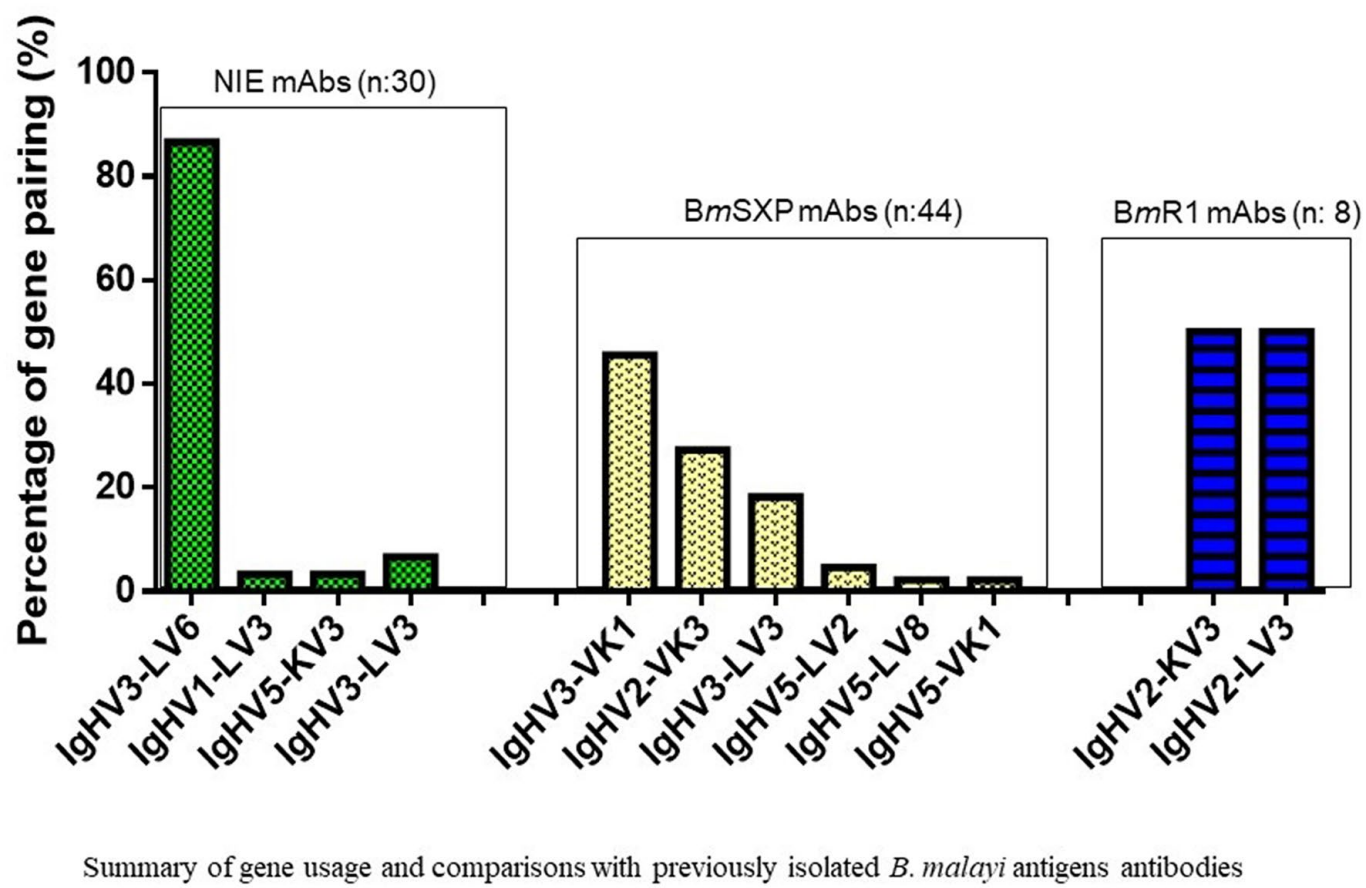
Analysis of gene pairing frequency of isolated of rNIE antigen antibody clones and comparison against previous *B. malayi* antigens antibodies (B*m*R1 and B*m*SXP).

The 30 scFv clones showed variations in their antibody gene family distributions clustered into 4 unique gene pairings (Fig. 2). In total, 29 out of 30 (97%) sequences showed functional variable lambda (VL) genes with only a single (3%) sequence for variable kappa (VK) gene. In addition, there was a preference seen in the heavy chain (VH) gene usage with majority of the clones originating from VH3 (94%) followed by VH5 (3%) and VH1 (3%). On the other hand, LV6 (87%) was the dominant gene used followed by LV3 (10%) and KV3 (3%) for the light chain. Next, there was a clear enrichment for one heavy and light chain gene pairing. The most common pairing was IgHV3-LV6 (87%) followed by IgHV3-LV3 (7%), IgHV1-LV3 (3%) and IgHV5-KV3 (3%). A comparison of the antibody gene family usage for *B. malayi* antigens is included and described in the discussion.

### Antibody sequence analysis

**Figure 3:**
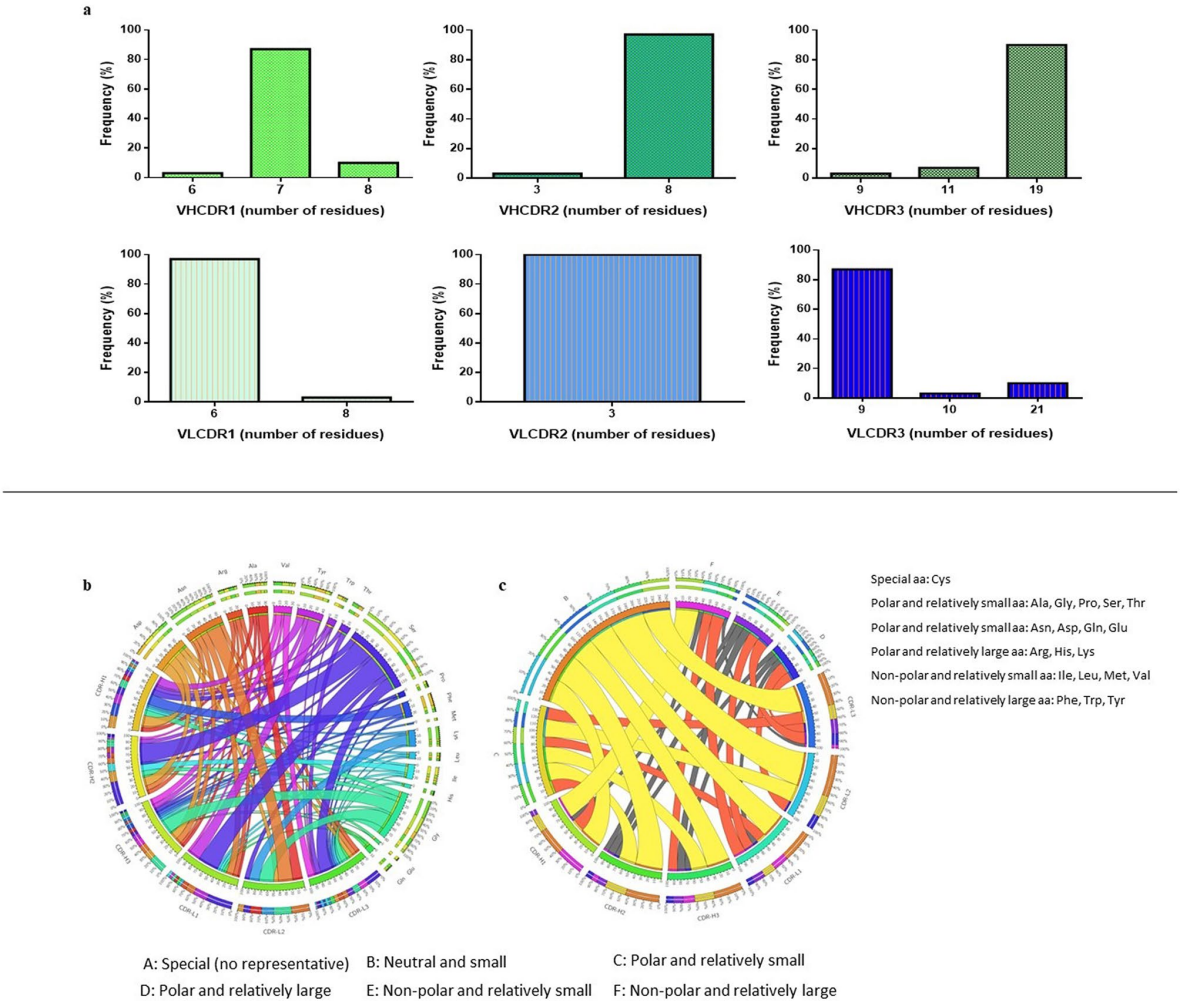
Monoclonal antibody clone characterization. (**a**) Amino acid length variations of heavy and light chains of antibody clones. (**b**) CIRCOS diagram analysis for amino acid frequency. (**c**) CIRCOS diagram analysis for amino acid polarity. CIRCOS diagrams were drawn using Circos Table Viewer v0.63–9 (http://mkweb.bcgsc.ca/tableviewer/visualize).

The CDR analysis consisting of the length and composition of the CDR region was observed due to its vital role for the binding site topology. The range of amino acid (aa) length for the VH is between 3 to 19 aa (Fig. 3a). The HC CDR1 has a length distribution from 6 to 8 aa with 7 aa being the dominant length. CDR2 had only 3 and 8 aa, whereas for CDR3 the distribution was 9, 11 and 19 aa. The dominant length for CDR2 and CDR3 was 8 aa and 19 aa, respectively. The distribution of the LC CDRs was also varied with 6 and 8 aa for CDR1 and 3 aa for CDR2. However, the length range for CDR3 was broader from 9 to 21 aa with 9 aa as the dominant length.

The aa propensity for the enriched scFv CDR regions was observed and a Circos plot analysis was performed to highlight the distribution patterns in a clearer presentation. It showed some regions with random aa distributions while others showed a skewed aa representation for both heavy and light chains (Fig. 3b). The Circos plot allows the visualization of the frequency of the aa present in each of the CDR. The HC CDR1 showed a higher representation of aspartic acid, phenylalanine and tyrosine compared to other aa, whereas the HC CDR2 showed over representation of serine. The highly diverse HC CDR3 was dominated with glycine, aspartic acid, valine and asparagine. Conversely, for LC CDR1 and CDR3 the distribution of aa was random but with a significant increase in serine while the LC CDR2 showed a skewed pattern of distribution with higher representations of glycine and asparagine. The unique presentation by the Circos plot allows for a global view to analyze relationship patterns between the different CDR. Figure 3b shows the dominance of serine in CDR-H2, CDR-L1 and CDR-L3. However, glycine was dominant in CDR-H3 and CDR-L2. The aa usage in CDR-L2 is unique in comparison to the other CDR with a more even distribution of lysine, glycine, asparagine and alanine. The aa usage in the other CDR are randomly distributed with the utilization of more different aa.

The Circos plots analyzing the polarity distribution for all CDRs in both chains showed similar distribution patterns with a higher representation of neutral and small aa in all CDR (Fig. 3c). CDR-H1 has a similarly high presence of non-polar and relatively large aa. CDR-H2 and CDR-L2 also has a significant amount of polar and relatively small aa present in the binding pocket. Moderate amounts of non-polar and relatively large aa is found in CDR-H3 and CDR-L1. Non-polar and relatively small aa are moderately distributed in CDR-L1 and CDR-L3.

In addition, the Kabat numbering scheme was used to further analyze the CDR3 aa position. CDR3 HC was determined to be from position 93 to 102, while position 89 to 97 was designated for CDR3 LC (Fig. S2). The dominance of certain amino acids can be observed at different positions. For CDR3 HC, at position 93 the dominating aa is alanine, 95 and 100C is asparagine, 97, 99, 100D and 100H is glycine, 100A and 100F is phenylalanine while position 100B and 100G was only restricted to tyrosine. The dominating aa at position 100E and 100I was only restricted to valine and methionine, respectively. For CDR3 LC, positions 95B, 95E and 95F were restricted to histidine, phenylalanine, and glycine respectively. Serine was dominating at position 90, 91, 93, and 95. At position 89 the dominating aa is glutamine, 92 is aspartic acid and 95A is asparagine.

### Structural analysis

**Figure 4:**
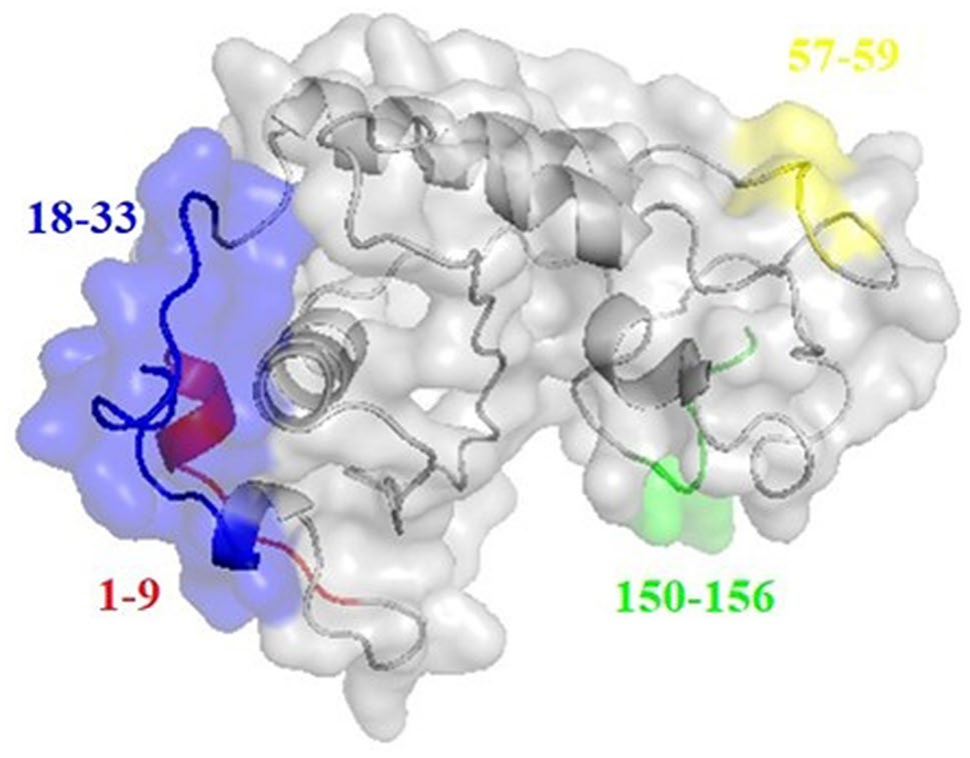
The four predicted consensus B-cell epitopes of NIE (residues 1–9, 18–33, 57–59 and 150–156). Figure was prepared by PyMol ver 2.4.0 (https://pymol.org/2/).

**Figure 5:**
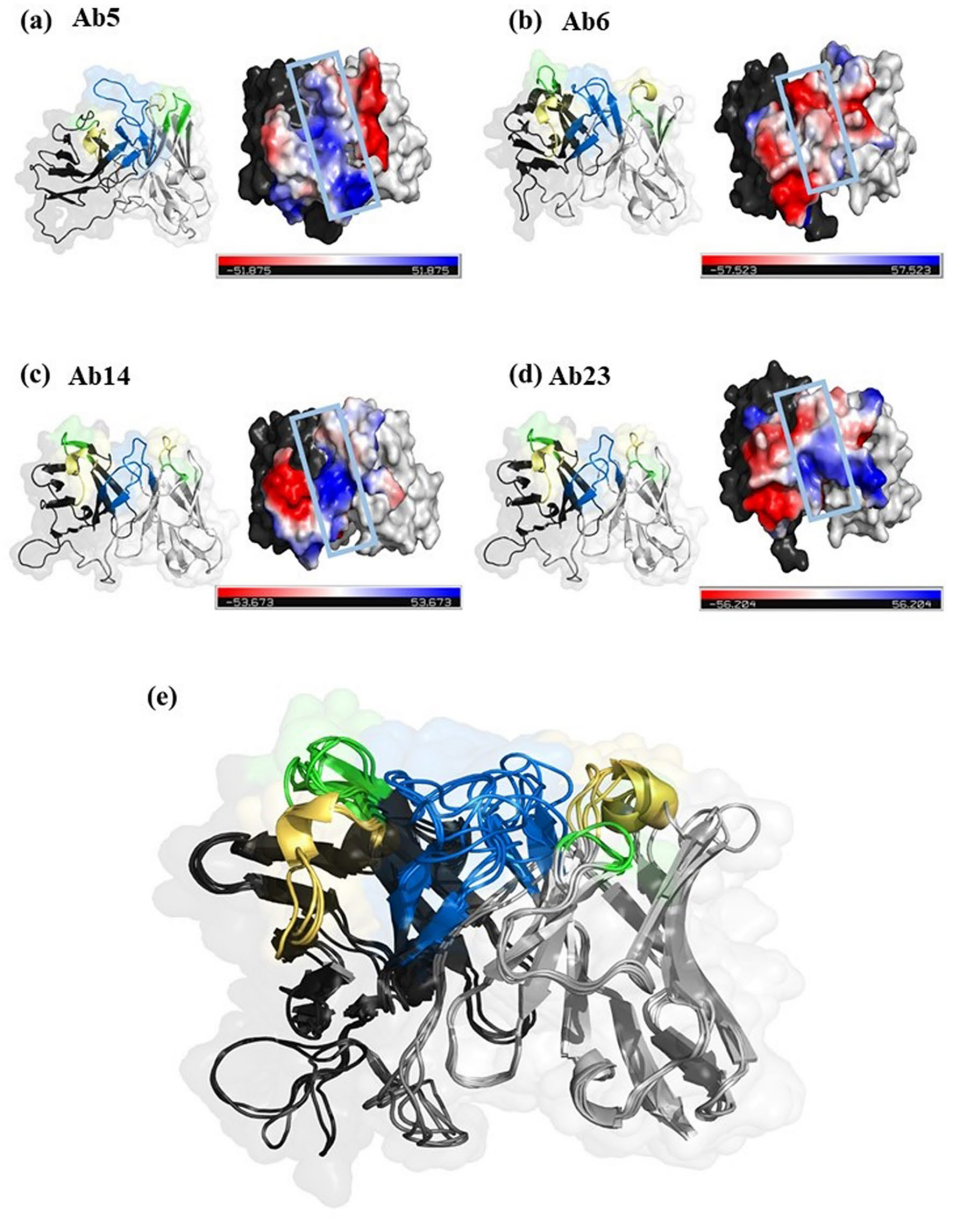
Three dimensional structures of rNIE specific antibodies (**a**) Ab5, (**b**) Ab6, (**c**) Ab14 and (**d**) Ab23, by surface/ribbon (left) and electrostatic (right) presentations. Variable light (VL) and variable heavy (VH) domains are in black and grey ribbon presentation, respectively. CDR1, CDR2 and CDR3 are in yellow, green and blue ribbon presentation, respectively. Electronegative and electropositive residues are in red and blue surface presentations viewed from top. CDR3 is highlighted with blue box. (**e**) Superimposition of the four modelled scFvs. Figure was prepared by PyMol ver 2.4.0 (https://pymol.org/2/).

The consensus secondary structure analysis showed that the N-terminal (residues 3–29) of the NIE is a helix while the rest of the region is coils/turns (Table S1). The secondary structure calculations of the NIE structures modelled by different approaches showed similar findings but with the addition of three, one two helices in the comparative, de novo and ab initio models, respectively (Table S2). Structural visualization on the NIE structure modelled using different approaches also showed a similar globular shaped protein and the structural stoichiometry evaluation of the comparative model has the best scores among the structures build from different approaches (Fig. S3). Therefore, the comparative model was selected for further docking simulation with scFvs. B-cell epitope prediction results indicated 4 possible epitopes for NIE (residues 1–9, 18–33, 57–59 and 150–156; Fig. 4). These 4 epitopes were then marked as the active interface residues for the docking simulation with the four antibody clones. On the other hand, the electrostatic energy analysis of the modelled antibody clones showed that Ab5 and Ab14 have more electropositive CDR3 while the CDR3 of Ab6 is more electronegative and CDR3 of Ab23 consists of both electropositive and electronegative residues (Fig. 5).

**Figure 6:**
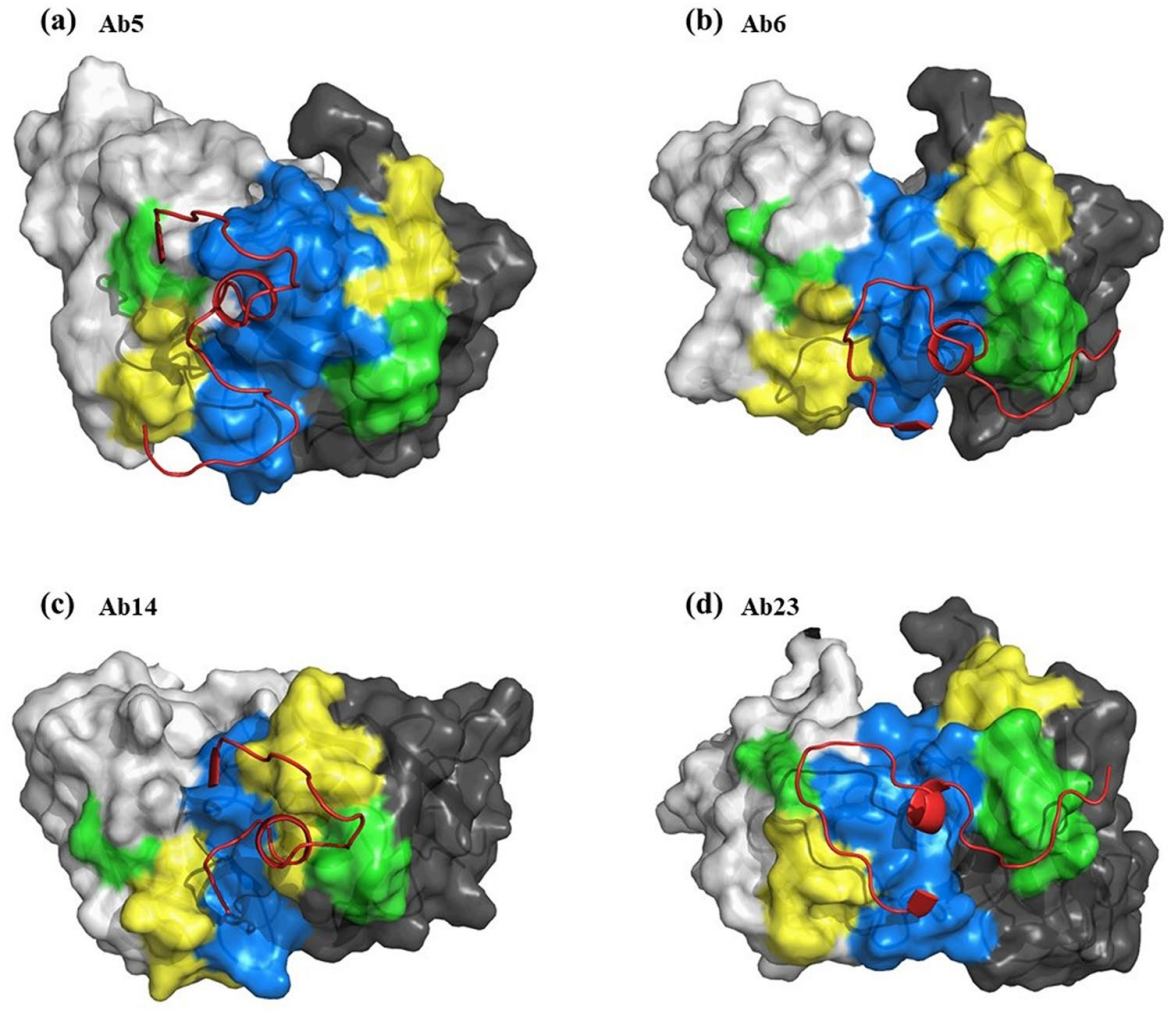
Surface presentation of the docked conformation of the antibody clones (**a**) Ab5, (**b**) Ab6, (**c**) Ab14 and (**d**) Ab23 with rNIE. VH and VL domains are in black and grey presentation with respective Yellow, green and blue represent CDR1, CDR2 and CDR3 respectively. Red ribbon is the rNIE residues that interacts with the antibody. Figure was prepared by PyMol ver 2.4.0 (https://pymol.org/2/).

Docked conformation of the antibodies showed different binding orientations with the NIE (Fig. 6). CDR3 was observed as the core antigen binding domain. Table S3 showed that all clones interact with NIE with the CDR3 except for Ab6. Ab14 and Ab23 have the highest number of interacting residues (36 and 32 interaction, respectively) with NIE compared with Ab5 and Ab6 (both with 26 interactions with NIE). The isoelectric point (pI) of Ab6 is 5.2 while other clones have pI values of more than 8.0 (Table S4). Therefore, Ab6 is the only clone with a negative net charge at pH7. The calculated interface area of Ab6 was also the lowest (614.4 Å) among the antibody clones. Estimated binding free energies (G_Bind_) of the antibodies with NIE showed that Ab14 and Ab23 (G_Bind_ − 7.8 and − 6.1 kcal/mol, respectively) are better binders compared with Ab5 and Ab6 (both with G_Bind_ − 5.6 kcal/mol).

IMGT Colliers de Perles was also applied to analyse the structure of the scFv variable domains depicted with a unique numbering system and standardized 2D graphical representations of the antibody molecule (data not shown). This tool uses statistical methods to analyze the aa and hierarchic classification of framework positions based on three properties (hydropathy, volume and physiochemical characteristics). The analysis consists of the distribution of hydrophobic aa, hydropathy and other aa properties displaying the key aa that play vital role in protein interactions and domain structures. Based on 2D graphical representations, all four antibody clones showed similar pattern of aa distribution.

### Preparation of recombinant monoclonal antibody protein

**Figure 7:**
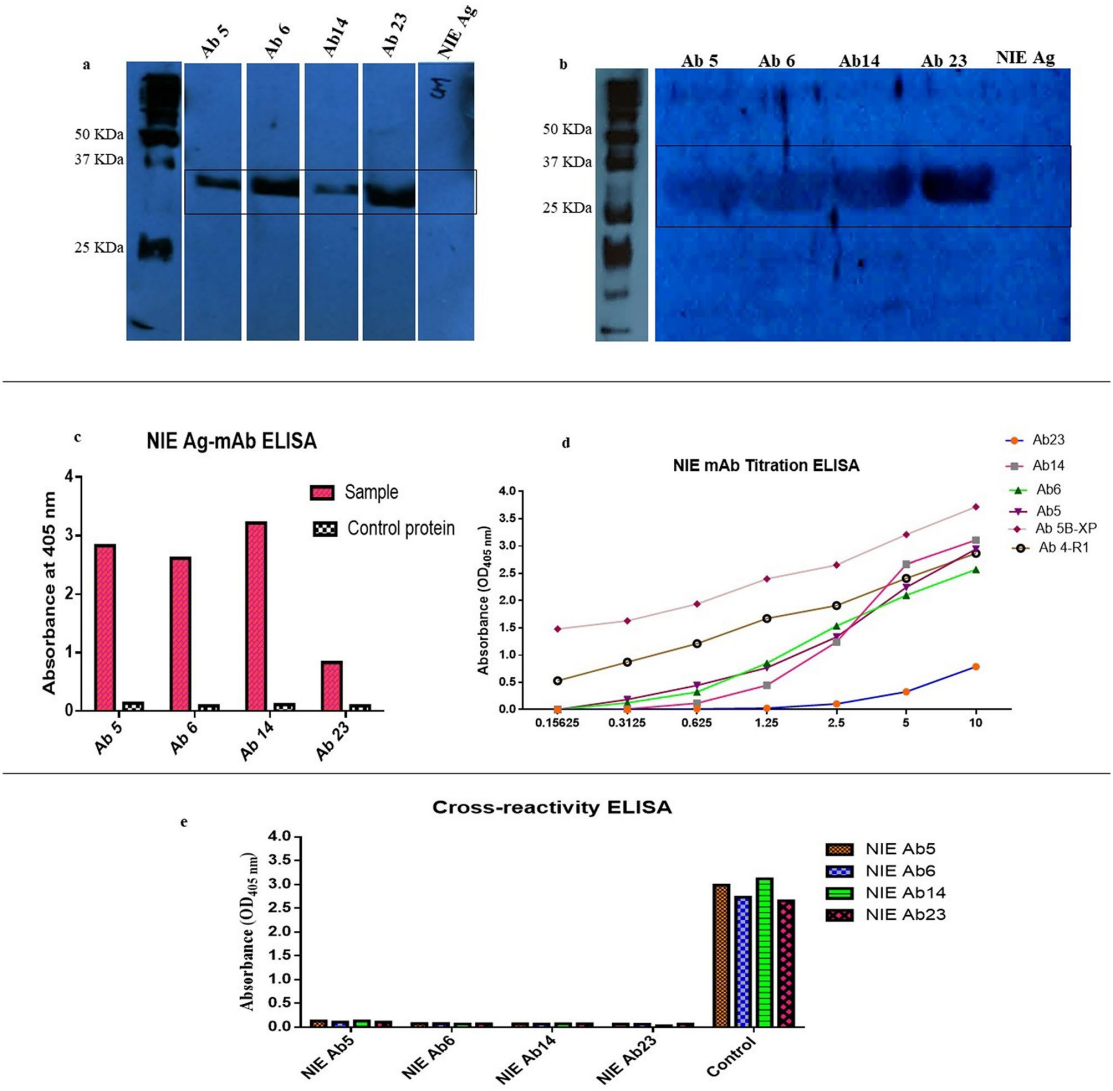
Verification and binding analysis of rNIE specific recombinant monoclonal antibody proteins. (**a**) Western blot analysis of recombinant monoclonal antibody proteins. (**b**) Antigen–antibody Western blot analysis. (**c**) Immunoassay analysis of recombinant monoclonal antibody proteins. (**d**) Antibody titration ELISA; Ab23, Ab14, Ab6, Ab5 are antibody clones against rNIE, Ab5B-XP and Ab4-R1 are antibody clones against BmSXP and BmR1 antigens respectively (**e**) Cross-reactivity ELISA. All the binding verifications were detected using Strep Tactin HRP conjugated antibody except for cross-reactivity ELISA was detected using anti-M13 HRP conjugated antibody.

In total, four clones of unique gene families (Ab5: IgHV3-LV1; Ab6: IgHV3-LV6; Ab14: IgHV5-KV3; Ab23: IgHV3-LV3) were selected and successfully subcloned into pET 51(b) + vector for expression in SHuffle T7 *Escherichia coli* cells. The SDS PAGE (Fig. S4) and Western blot showed the expected molecular mass of the recombinant antibodies at approximately 35 kDa (Fig. 7a). The antibodies were expressed and purified at satisfactory levels using the SHuffle T7 *Escherichia coli* cells.

### Characterization of antigen–antibody binding

The antigen–antibody binding was characterized using four different experiments. First, it was determined using antigen–antibody Western blot (Fig. 7b), native antigen–antibody Western blot (Fig. S5) and antigen–antibody ELISA (Fig. 7c) and native antigen–antibody ELISA (Fig. S6). These assays were performed using Strep Tactin-HRP conjugated antibody which confirms the binding of the recombinant monoclonal antibodies to the target antigen. All the identified antibody clones were able to specifically bind to the target protein but with different band intensities. The Western blot band intensity of antigen–antibody Western blot was quantified using the ImageJ software (http://rsb.info.nih.gov/ij/). Ab23 showed the strongest band intensity with 44.5% followed by Ab14 (23.5%), Ab5 (19.4%), and Ab6 (6.3%).

Western blot and ELISA analysis using the native antigen was also carried out. It was performed to determine the binding capabilities of the newly isolated antibody clones to the native antigen. All four recombinant antibody clones showed binding to *S. stercoralis* native antigen (Figs. S5 and S6). Subsequent analysis was performed using titration ELISA to determine the limit of binding of the antibody clones. The highest binding strength at lowest protein concentration was shown by Ab5 followed by Ab14, Ab6 and Ab23 (Fig. 7d). The range of recombinant monoclonal antibody protein concentration was 10 μg to 0.156 μg/well. Ab5 was able to bind as low as 0.625 μg/well, followed by Ab14 and Ab6 with a binding limit of 1.25 μg/well, and Ab23 at 2.5 μg/well. In addition, the limit of detection of previously isolated recombinant monoclonal antibodies against *Brugia malayi* antigens using the same HAYDEN Filariasis library was included. Ab 5B-XP and Ab 4-R1 are recombinant monoclonal antibodies against BmSXP and BmR1 antigens respectively. BmSXP and BmR1 specific antibodies showed highest binding limits as both antibodies able to bind as low as 0.156 μg/well. Cross-reactivity analysis using ELISA was also carried out. The profiles showed that all four antibody clones were competing for the same epitope on the target antigen highlighting a predominant epitope with high immunoreactivity (Fig. 7e). For all three types of ELISAs performed, the absorbance values of the background protein control were subtracted from the readings of the samples.

## Discussion

Generally, phage display antibody libraries from natural and synthetic repertoires have been useful sources for the generation of antibodies against various targets of interest for medical and research applications^17^, and some libraries have been developed into technological platforms^18^. One of the key factors for this success is the robustness of the bacteriophage to display diversified antibodies that allows an expanded coverage for a diverse selection of target antigens. The increased understanding of antibody-antigen binding properties has also enabled improved strategies in antibody generation.

The HAYDEN-Filariasis library used in this study is an immune antibody phage display library constructed using LF blood donors^6^. LF is mainly caused by *Wuchereria bancrofti* and *Brugia malayi*, while *S. stercoralis* is the main cause of strongyloidiasis. Both helminth diseases share similarities in terms of immune responses to the parasites. The target used in this study was rNIE, a specific and sensitive protein for detection of *S. stercoralis* infection that can be produced with high yield and good purity^16^. Previously, the HAYDEN-Filariasis immune library was successfully utilized to produce antibodies against two important filarial antigens, BmSXP and BmR1^6,19^.

An immune antibody library is designed to represent a biased collection of antibody repertoires due to the exposure of the host to a particular pathogen. Therefore, the host antibody repertoire is mainly enriched with disease-specific antibody clones that have undergone affinity maturation processes such as clonal selection and somatic hyper mutation. This increases the likelihood of enriching high affinity antibodies against antigens of the targeted disease. However, when considering the essence of phage antibody library cloning, it is likely that the combinatorial amalgamation of antibody genes will inadvertently invoke unnatural gene combinations to materialize. This has an unprecedented consequence to the way immune library applications are perceived. The additional repertoire generated by unnatural gene combinations can expand the coverage of an immune library to enrich antibodies for non-targeted disease-related antigens. However, the immediacy of predominant target disease-skewed antibody gene repertoire is expected to permit a limited spectrum of non-disease antigens to be covered by the repertoire. Nevertheless, it is conceivable that antigens of closely related diseases to the primary disease of the immune library repertoire could be targeted for antibody generation by using the initial immune library. This is exemplified in a recent study which reported the use of an immune library for the isolation of antibodies against various non-immune antigens^20^. This is likely due to the presence of large amounts of unimmunized antibody clones, together with biased immunized clones in an immune library repertoire. Hence, it is not surprising that an immune library may be diverse enough to function like a naïve library and be applied to target various antigens. The presence of unimmunized clones emphasizes the broadness of the B-cell memory repertoire in the immune system.

Subsequently, another possible consideration would be the choice of sample collection during immune library constructions. The stage at which patient samples are collected can range from acute, recovering to recovered from an infection. The antibody configuration will vary as a result of the different immune response at the different stages of infection, much like the distribution of different immunoglobulin isotypes in a disease cycle. Therefore, it is conceivable that a sample antibody composition contains a mixed population of activated B cells during encounter with an antigen and also unexposed antibody clones which are naïve B cells. The HAYDEN-filariasis library used in this study consists of blood from apparently asymptomatic individuals who had high density of circulating microfilariae, i.e. ~ 2600–15,000 larvae per ml of blood. This category of individuals would generally display a broader range of antibody responses compared to symptomatic or asymptomatic individuals with low infection load^21^. In general, intestinal nematode parasites usually stimulate CD4 T cell dependent Th2 type polarized immune responses with potentially overlapping effector mechanisms^22^.

BLAST analysis of the NIE rAg sequence against *B. malayi* sequences was performed in order to determine the extent of sequence identity. At the nucleotide level both sequences showed no identity. Meanwhile at the protein level there was some similarities ranging from 26.23 to 48.28%. ClustalW alignment was performed to show the conserved and non-conserved regions. This is attached as supplementary file as Fig. S7. The homology between *B. malayi* genome and NIE protein is expected as they are both nematodes. Although low, the percentage of similarity may also explain the ability of the HAYDEN-Filariasis immune library to isolate antibodies against the NIE protein from *S. stercoralis*. This possibility cannot be discounted as a potential reason for the antibody library repertoire to isolate antibodies against proteins from other nematodes.

In total, 30 full length antibody clones representing four unique gene families were identified. It is noticeable that 97% of the isolated clones were dominated by functional variable lambda genes and only 3% of sequences were kappa genes. The preferred V gene family was VH3 for HC and VL6 for LC and the most common gene pairing was IgHV3-VL6. Conversely, antibodies to B*m*SXP filarial antigen (previously isolated from the same library) showed more functional variable kappa genes compared to functional lambda genes whereas antibodies to B*m*R1 filarial antigen (also previously isolated from the same library) showed equal representation of variable kappa and lambda genes. The V-gene preference for antibodies raised against the two filarial antigens using the same library showed different gene usage. The B*m*SXP antibody clones were VH3, similar to that of NIE, while BmR1 antibody clones were mainly represented by VH2. Overwhelming predominance of VH3 antibodies for defence against a variety of bacteria and viruses have been previously reported^23–25^. The most common gene pairings for LF were IgVH3VK1 for B*m*SXP and IgVH2VL3 and IgHV2-VK3 for BmR1. This is thus different with rNIE whereby VL6 was dominant.

The gene encoding the kappa gene segments are located on chromosome 2^26^ while lambda gene segments are encoded on chromosome 22^27^. Although there are more kappa antibodies in the human peripheral blood, in antigen-selected populations it can vary significantly depending on the class of antibody heavy chain^28^. Furthermore, over-representation of the lambda or kappa subfamilies is mainly based on the interaction of the antibody gene segments during antigen encounter. Many studies have reported the dominance of lambda subfamilies compared to kappa subfamilies in different infections^29–31^. For example, antibodies from the mucosal region^32^ and HIV-specific antibodies^33^ showed a strong bias of enriching lambda antibodies. It is because lambda antibodies are more stable when paired with different VH families to produce more stable scFv antibodies. The stability is due to its higher scFv-pIII fusion protein expression levels. In addition, kappa antibodies; showed poor expression in *E. coli* compared to lambda antibodies^34^.

Complementary determining region (CDR) loops are the most diverse region of an antibody. The length of the CDR determines the topological variation of the binding sites^35^. The range of CDR length for the HC was 3 to 19 amino acids and LC was 3 to 21 amino acids. The length variations can contribute to the information of the binding site motifs^36^. For example, deep pocket motif antibodies tend to bind haptens, while peptide binding antibodies usually appear as grooves, and antibody that binds to protein is normally flat^37^. The CDR-H3 plays the most important role during antibody-antigen binding and the average length of this loop is around 1 and 35 amino acid in a pattern resembling a Gaussian distribution^38^.

In addition, antibodies raised against the two filarial antigens using the same library showed some variation in terms of CDR length^6,19^. For heavy chain, the CDR1 length was similar for all three antigens (NIE, BmSXP and BmR1) which was around 6–10 aa. NIE antigen showed similarities with BmSXP antigen for CDR2 and CDR3 regions. The CDR1 ranges from 3–8 aa and CDR3 ranges from 9–20 aa. Conversely for BmR1 the CDR2 and CDR3 lengths were 3 aa and 9–11 aa. Subsequently, light chain CDR1 length for all three antigens was about 6–10 aa. The CDR2 of the NIE showed the shortest length with 3 aa compared to both filarial antigen that was in the range from 7–8 aa.

The docked complex of NIE-Ab in this study has further evidenced the interactions between the antigen and antibody is mainly at the CDR3. Long CDR-H3 are usually associated with autoreactivity as it would have been selected against the target as B cell matures and successfully pass through tolerance checkpoints^39,40^. However, the role of CDR-L3 should not be overlooked as the physiochemical and structural differences of this region reflect different roles in the immune response^28^. The stability of the longer CDR’s are internally stabilized through the presence of cysteine forming disulfide bridges.

Subsequently, the amino acid usage and polarity of NIE mAbs against previously isolated Filarial antigens mAbs are comparable with some variations. For all the mAbs the aa at CDR3 regions showed highest variations. However, representations of aa were different, for B*m*R1 antigen the heavy chain of CDR3 was dominated by arginine and alanine. On the other hand, BmSXP antigen showed random equal distribution of all aa but slightly higher serine. Meanwhile for NIE antigen the mAbs showed over representation of glycine, aspartic acid, valine and asparagine. Heavy chain of CDR2 region of BmSXP and NIE showed over representation of serine while BmR1 showed higher aspartic acid. Heavy chain of CDR1 though with the presence of other aa but both filarial antigens showed dominancy of serine and glycine while NIE antigen showed over representation of aspartic acid, phenylalanine and tyrosine. Interestingly for light chain of CDR1 and CDR3 despite with the presence of other aa all three antigens showed a significant increase in serine. CDR2 of the light chain for BmR1 showed abundance of glycine followed by BmSXP with proline and serine meanwhile NIE showed higher representation of glycine and asparagine. Looking at the polarity of all the three antigens, in total all showed higher representation of neutral and small aa due to rich in aa such as serine and glycine.

By using the crystal structure of Ves v 2^41^ as the template in comparative modelling, the built NIE 3D structure has the best evaluation scores for its global stereochemistry compared with the structures predicted other approaches. This could be due to NIE and Ves v 2 having sequence similarity of nearly 40%. In addition, the experimental data also showed that NIE shares epitope with Ves v 5^41^. The physiochemical properties calculations of Ab6 with the lowest interface area and the only negatively charged antibody at pH7 could have explained the weakest intensity of Ab6 band observed in the Western blot. The antibody clones were further analysed using IMGT Colliers de Perles tool. The bioinformatic analysis of the CDR regions permits the visualization of the aa which are important for the 3D structural configuration and to delineate the standardized framework regions. For example, the length of the helix represented in IMGT Colliers de Perles becomes a crucial information in the domain characterization and are particularly useful for sequence-structure analysis, antibody engineering, visualization and comparison of positions for mutations, polymorphisms and contact analysis^42^. This structural information would be useful for antibody refinement in the future studies.

All four recombinant monoclonal antibody proteins were successfully expressed, purified and the binding analysis conducted confirmed the specificity towards the NIE target antigen. An *in-house* unrelated protein control carrying the same tags as rNIE was used when performing all types of ELISA. In addition, the expression and purification tag for monoclonal antibody proteins in this study was anti-His. However, the detection tag for polyclonal and monoclonal ELISA was anti-M13 horseradish peroxidase (HRP) and for binding assays was Strep Tactin- HRP. Different tags were used for detection in order to ensure correct protein detection.

The titration ELISA showed Ab5, Ab6 and Ab14 exhibiting approximately similar binding strength while Ab23 showed lowest strength of binding. The native antigen Western blot also showed the capabilities of all four antibody clones binding the *S. stercoralis* native antigen. Furthermore, cross-reactivity ELISA shows the ability of all four monoclonal antibody clones with different V-gene family usage and light chain pairing competing for a similar epitope. This is evidence of the diversity and flexibility of antibody molecules to adapt different structural motifs to bind to a similar epitope. This results in the presence of different antibodies against a single epitope that affects the evolution of the pathogen for host recognition. In addition, multiple antibodies targeting one binding site of an antigen allows for rapid recognition and destruction by other components of the immune system. In the present study, the presence of multiple antibodies against a single dominant epitope highlights the immunoreactivity of the epitope for the rNIE.

Existing commercial diagnostics to detect *Strongyloides* infection in blood/serum/plasma are antibody-detection assays. These assays are sensitive but may detect past or treated infections and may not detect early infection. Antigen detection assay can address the above limitations, thus it can complement existing antibody-detection tests. There are only a few reports on antigen detection for strongyloidiasis, and one of them used recombinant antibody to *Strongyloides* HSP60 protein^43^. The anti­HSP60 scFv fragment was reported to detect immune complexes in strongyloidiasis patients’ serum samples in an ELISA. Subsequently, in a follow-up study, the ELISA detected six times (27.27%) more positive samples as compared with conventional parasitological techniques (4.54%)^44^. In the future, the recombinant antibody to rNIE from the present study can be used to develop an antigen detection assay; subsequently, the diagnostic sensitivity and specificity can be compared with reported antigen-detection ELISA.

In conclusion, the present study provides a small glimpse into the *Strongyloides*- specific antibodies derived from non-target disease specific antibody library. This study also highlights the depth of the antibody repertoire even from a skewed population to yield antibodies against a disease target that was close to the parent disease where the library repertoire was derived from. The sequence-structure analysis was deciphered in order to characterize the newly isolated antibody clones. Analysis of the antibody physiochemical properties highlighted the positively charged molecule at pH7 and interface area could determine the amount of bound antibody with NIE observed in the Western blot. In future, further structural analysis especially epitope mapping of the antibody clones is required to elucidate the underlying interactions and identify the key epitope of NIE. It is also important to note that the antibody clones identified in this study are potentially useful for various biomedical applications in the near future.

## Methods

### Recombinant protein preparation

A single colony of bacteria expressing the rNIE was inoculated into 100 mL Terrific broth (TB) media containing 100 μg/mL ampicillin, and cultured overnight at 37 °C, 200 rpm. The next day, 10 mL of the overnight culture was inoculated into 4 × 500 mL TB supplemented with 100 μg/mL of ampicillin and incubated at 37 °C with agitation at 200 rpm until OD_600_ of 0.6–0.8 was reached. Subsequently, 0.5 mM of isopropyl-B-d-thiogalactopyranoside (IPTG) was added into the culture and incubated for 4 h at 28 °C. The culture was harvested by centrifugation at 10,000×*g* for 10 min at 4 °C. The cell pellet was resuspended in cold lysis buffer (50 mM NaH_2_PO_4_, 500 mM NaCl and 10 mM imidazole) containing lysozyme (0.5 mg/mL) and protease inhibitors. The mixture was incubated on ice for 30 min. The mixture was then lysed mechanically using a French press (1500 psi, 1 min). The lysate was then centrifuged at 10,000×*g* for 5 min at 4 °C to separate the supernatant from the cell debris. *DNa*se I was added at a concentration of 0.5 μg/ml to the supernatant and incubated on ice for 15 min. This was followed by another round of centrifugation at 10,000×*g*, 4 °C for 30 min. The supernatant was then collected and filtered through a 0.45 μm filter membrane to remove debris that may block the purification column. The filtered supernatant was mixed with the Ni–NTA slurry resin (cOmplete His-Tag purification resin, Roche, Switzerland). The resin-lysate mixture was incubated at 4 °C for 1 h with gentle rotation. The wash column was preloaded with resin-lysate mixture and washing was performed using wash buffers containing 20 mM, 30 mM, and 40 mM of imidazole. The target protein was eluted using the elution buffer containing 250 mM imidazole and the fractions were analysed through SDS-PAGE. The protein-containing fractions were pooled, and buffer exchanged into phosphate-buffered saline (PBS, pH 7.4) using Amicon Ultra 10 mL centrifugal filter (Merck, Germany). The presence of the target protein was confirmed via Western blotting using the anti-His6-tagged secondary antibody at 1:3000 dilution. All chemicals used are from Merck (Germany) unless stated otherwise.

### Biopanning

Biopanning was carried out as previously reported^6^ using a human helminth scFv phage display library (HAYDEN-immune filariasis) against the rNIE. The construction of the library was previously described and have been applied for the isolation of scFv clones against other target antigens^6,19^.

### Phage ELISA DNA sequencing

Polyclonal and monoclonal phage ELISA were performed as previously reported^6^. The positive clones from the monoclonal ELISA were grown at 37 °C and 200 rpm, overnight (~ 16 h). The cell pellets were harvested, and plasmids were purified using QIAprep Spin Miniprep Kit (Qiagen, Germany), then sent for sequencing (FIRST Base Laboratories Sdn Bhd, Malaysia). The sequencing results were analyzed using IMGT/V-QUEST bioinformatics tool available at IMGT, the International ImMunoGeneTics information system^45–47^. An *in-house* unrelated protein control that can be detected using anti-M13 HRP was included as positive control and for background control the wells were coated with BSA.

### Recombinant monoclonal antibody protein expression, purification and verification

Plasmids of positive clones from monoclonal phage ELISA were subcloned into pET 51(b) + vector for expression in SHuffle T7 *Escherichia coli* cells (NEB, USA) and expression was performed in 1 L 2-YT broth supplemented with 100 μg/mL ampicillin and 0.2% glucose. The culture was grown at 37 °C, 200 rpm until the OD_600_ reached 0.6–0.7, then induced with 1 mM IPTG and further cultured for 16 h at 25 °C with shaking at 180 rpm. The pellet was harvested and resuspended in ice cold lysis buffer (50 mM NaH_2_PO_4_, 500 mM NaCl, 10 mM) containing imidazole and 0.5 mg/mL lysozyme). The mixture was then incubated on ice for 30 min and then subjected to cell breakage. It was performed using a sonicator for 4 cycles at 30 s/cycle with an output of 4.5 Hz. The disrupted cells were centrifuged at 10,000×*g* for 30 min at 4 °C. The supernatant was carefully collected and filtered through a 0.45 μm filter membrane.

The purification was carried out using C-terminal His-tag with a nitrilotriacetic acid (Ni–NTA) purification column (Qiagen, Germany) according to the manufacturer’s instructions. The purified proteins were then verified by SDS PAGE and Western blotting was performed using Strep Tactin-HRP conjugated antibody (1:3000). The bands were developed on film using Pierce ECL Plus substrate (Thermo Scientific, USA).

### Antigen–antibody binding assay

Antigen–antibody Western blot was carried out by running the rNIE on 12% SDS PAGE at 100 V for 1 h until the front dye reached the bottom of the gel. Then protein was transferred onto the nitrocellulose membrane using semi dry transfer at 12 V for 30 min. The membrane was then cut into stripes and incubated with the respective monoclonal antibody proteins at 4 °C overnight followed by incubation with Strep Tactin-HRP conjugated antibody (1:3000). The bands were developed on film using Pierce ECL Plus substrate (Thermo Scientific, USA).

For native antigen Western blot, the crude lysate antigen was first prepared. Briefly, a 50X protease inhibitor cocktail tablet (Roche, Switzerland) was dissolved in 2 mL of deionized water to produce a 25X protease inhibitor solution. A volume of 60 µL of the solution was added to 1.5 mL of *S. stercoralis* infective larvae and incubated on ice for 30 min*.* This mixture was then lysed by sonication on ice using a microtip probe for 3 min (30 s ON, 30 s OFF, 4.5 Hz). The lysed mixture was transferred to a cryovial and dipped into liquid nitrogen for 30 s (snap freeze) and then immediately thawed in a water bath for 20 min. The freeze-thawed mixture was centrifuged at 14,000 rpm for 5 min and the supernatant (soluble larval lysate) was collected. The protein concentration of the lysate was determined by RCDC Protein Assay (BioRad, USA) and stored at -20˚C until further use.

The native antigen Western blot was carried out similarly as the rNIE antigen–antibody Western blot as described above but with slight modifications. The modifications are 100 µg of *S. stercoralis* infective larvae protein was separated on SDS-PAGE and dilutions of the Strep-Tactin HRP conjugate used was 1:2000. Polyclonal anti-NIE was used as positive control and detected using goat-anti-rabbit HRP conjugate (1:5000).

Antigen–antibody ELISA was performed using rNIE and crude lysate antigens according to previously used method with slight modifications^19^. ELISA was carried out by coating 10 μg/well of rNIE or 50 µg/mL and 100 µg/mL of crude lysate antigens on Maxisorb ELISA microtiter plate (Nunc, New York, NY, USA) with 1XPBS, pH 7.4 overnight. The coated wells were washed with PBST (PBS with 0.5% Tween 20) and blocked with MPBST (2% skim milk in PBST) for 1 h at 37 °C, 600 rpm. After washing, each well was incubated with the respective monoclonal antibody proteins for 2 h at room temperature, 600 rpm. The plate was then washed and incubated with Strep Tactin HRP conjugate antibody (1:3000) for 1 h at RT, 600 rpm. The plate was washed three times with PBST and ABTS substrate was added and left to incubate in the dark for 30 min. The absorbance value was read at 405 nm using the SkanIT absorbance reader (Thermo Scientific, USA).

### Titration ELISA

Antibody titration ELISA was performed to determine the limit of binding of the identified anti-rNIE monoclonal antibody clones. The ELISA was performed similarly as described above but with one modification. After the second washing step, each well was incubated with the scFv protein at concentrations ranging from 10 μg/well to 0.156 μg/well.

### Cross-reactivity ELISA

Cross-reactivity ELISA was performed as previously reported^6^^.^ Briefly, the plate was coated with 10 μg/well of rNIE. Then the wells were washed, blocked, and incubated with 50 μg/well of the purified monoclonal antibody proteins. After 1 h, the wells were washed and 10^5^ of scFv phage was added accordingly and incubated for 1 h at room temperature (RT). The wells were then washed and incubated with 100 μL anti-M13 HRP conjugated antibody (1:5000 dilution) for 1 h at RT. Then the plate was washed and ABTS substrate was added. The absorbance value was measured at 405 nm using a SkanlT absorbance reader (Thermo Scientific, USA) after 30 min incubation in the dark. The absorbance values from the control wells were subtracted from test wells to obtain total absorbance.

### Structural analysis

The secondary structure of NIE protein was predicted using secondary structure prediction servers- PsiPred^48^, JPred4^49^ and Spider3^50^. The 3D structure of NIE was modelled via comparative modelling^41^, threading (ITasser)^51^ and DMPFold^52^ and ab initio (Robetta)^53^ approaches. The modelled structures from the above mentioned approaches were then evaluated for their compatibility using Procheck^54^, MolProbity^55^, Vadar^56^, Verify3D^57^, Errat2^58^ and PROVE^59^. The best model from the structural evaluation results was selected for docking with the scFvs.

On the other hand, the 3D model of the four scFvs (Ab5, Ab6, Ab14, Ab23) were built via comparative modelling (MODELLERv9.20; template PDB id 1RZI, 5WKZ, 4KQ3, 2DD8, 6RCQ, 5J66 for Ab5; 1W72, 3GIZ, 4QF1, 6MG4, 6E63, 4RIR for Ab6; 6MI2, 4KMT, 5WK2, 6GHG, 5I17, 6B9J for Ab14; 3GIZ, 1W72, 4QF1, 5BV7, 5Y9J, 4OD2 for Ab23). Physicochemical characteristics and electrostatic distribution for the scFvs were analysed prior to docking simulation by the jsPISA v.2.0.4^60^. The isoelectric point was calculated using Protein Calculator v3.3 (http://protcalc.sourceforge.net/; accessed 6th October 2019) and isoelectric point calculator^61^. Delphi program^62^ with atomic charges and radii parameters were set to AMBER force fields to calculate the electrostatic free energies and pKa of scFv CDRs titratable residues.

Molecular docking was applied to elucidate the interactions of the scFvs with NIE. Prior to docking simulation, the epitope for NIE was predicted by consensus results from three different independent predictions, namely BepiPred^63^, CBTope^64^ and Ellipro^65^. During docking simulation, only scFv CDRs residues and the predicted NIE epitopes were set as the interface residues. The docking simulation was performed using ClusPro Antibody mode^66^ available in ClusPro 2.0 server^67^. The scFv-NIE complex with the most negative binding free energy (∆G_Bind_) obtained from the most populated cluster with root mean square deviation (RMSD) tolerance of 2.0 Å was selected as the representative structure for further interactions analysis using jsPISA v.2.0.4. All scFv-NIE complexes were depicted using PyMOL molecular visualization system^68^.

The two-dimensional graphical representations of monoclonal antibody protein domains were constructed using IMGT Collier de Perles tool^69^. The CDR3 amino acid position and composition was further analysed using the Kabat numbering scheme and presented as heat map using heatmapper.ca online software^70^.

## Supplementary Information

Supplementary Information
