# Supplementary material for: Broad specificity of immune helminth scFv library to identify monoclonal antibodies targeting Strongyloides
Source: Sci Rep. 2021 Jan 28;11:2502. doi: 10.1038/s41598-021-82125-3 (PMC7843650; doi:10.1038/s41598-021-82125-3)
Supplement: Supplementary file 1 — Supplementary Information [file 41598_2021_82125_MOESM1_ESM.docx]

**Broad specificity of immune helminth scFv library to identify monoclonal antibodies targeting *Strongyloides***

Anizah Rahumatullah^1^, Dinesh Balachandra^1^, Rahmah Noordin^1^, Zamrina Baharudeen^1^, Yee Ying Lim^1^, Yee Siew Choong^1^ & Theam Soon Lim^1,2*^

^1^Institute for Research in Molecular Medicine (INFORMM), Universiti Sains Malaysia, 11800 Minden, Penang, Malaysia.


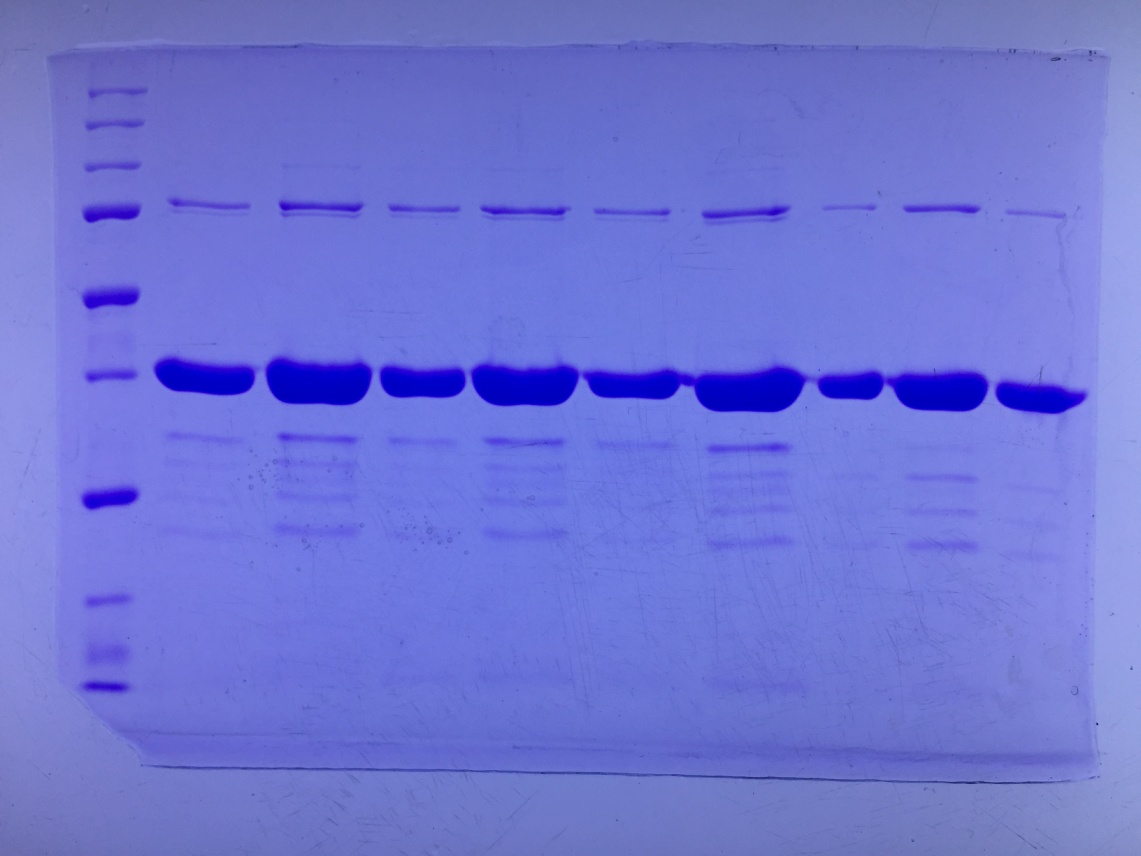

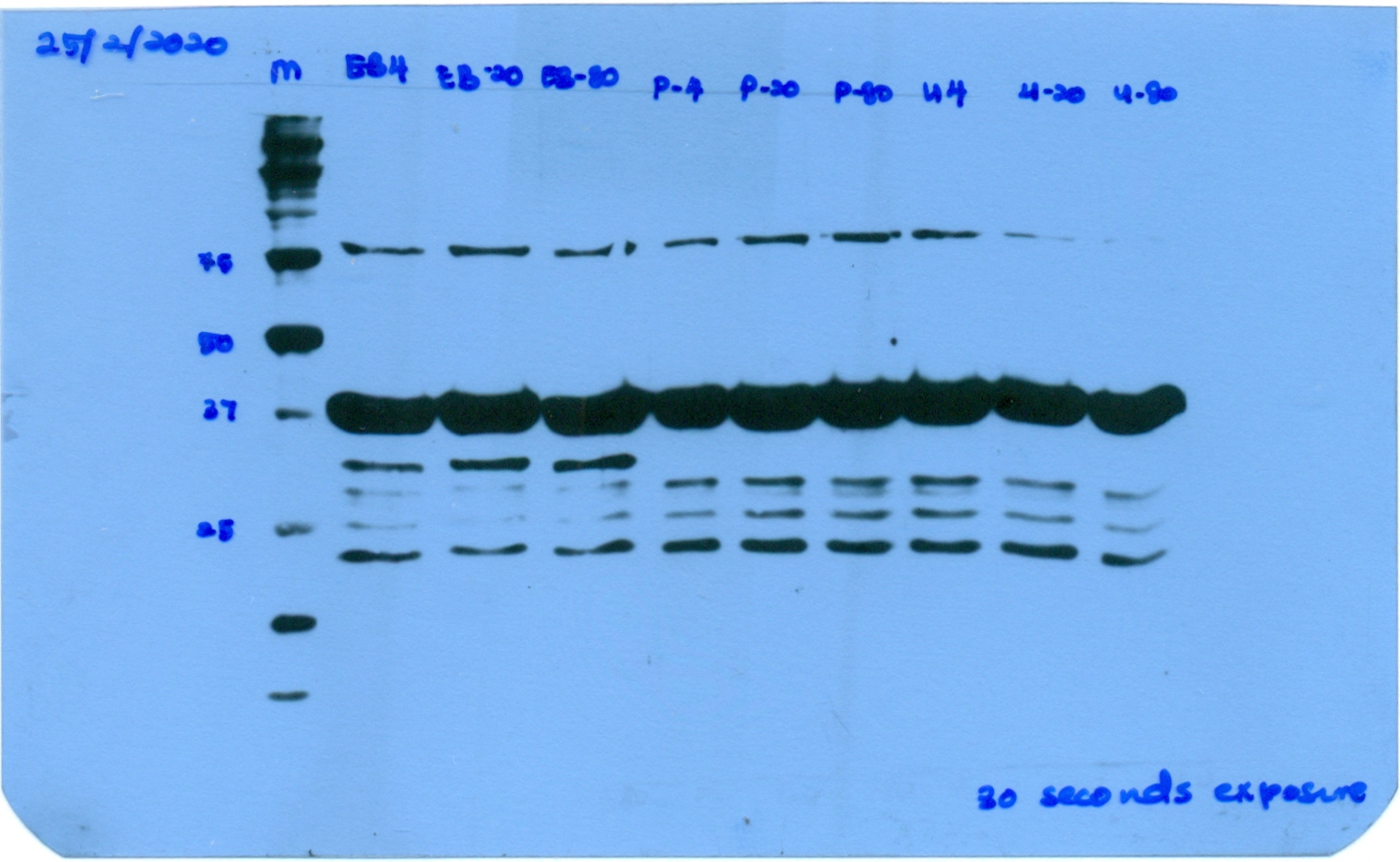
**a b**

50kDa

37kDa

30kDa

25kDa

15kDa

20kDa

50kDa

30kDa

25kDa

37kDa

c


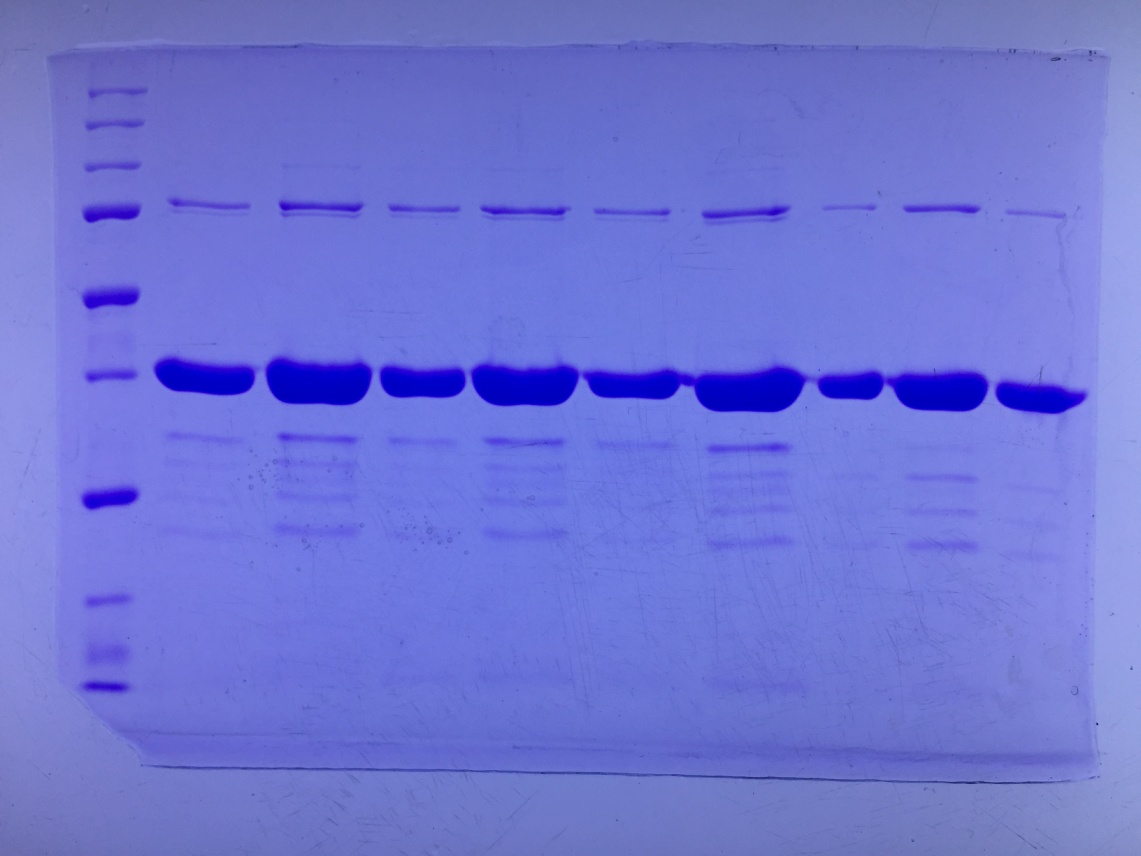


15kDa

25kDa

30kDa

37kDa

50kDa

d


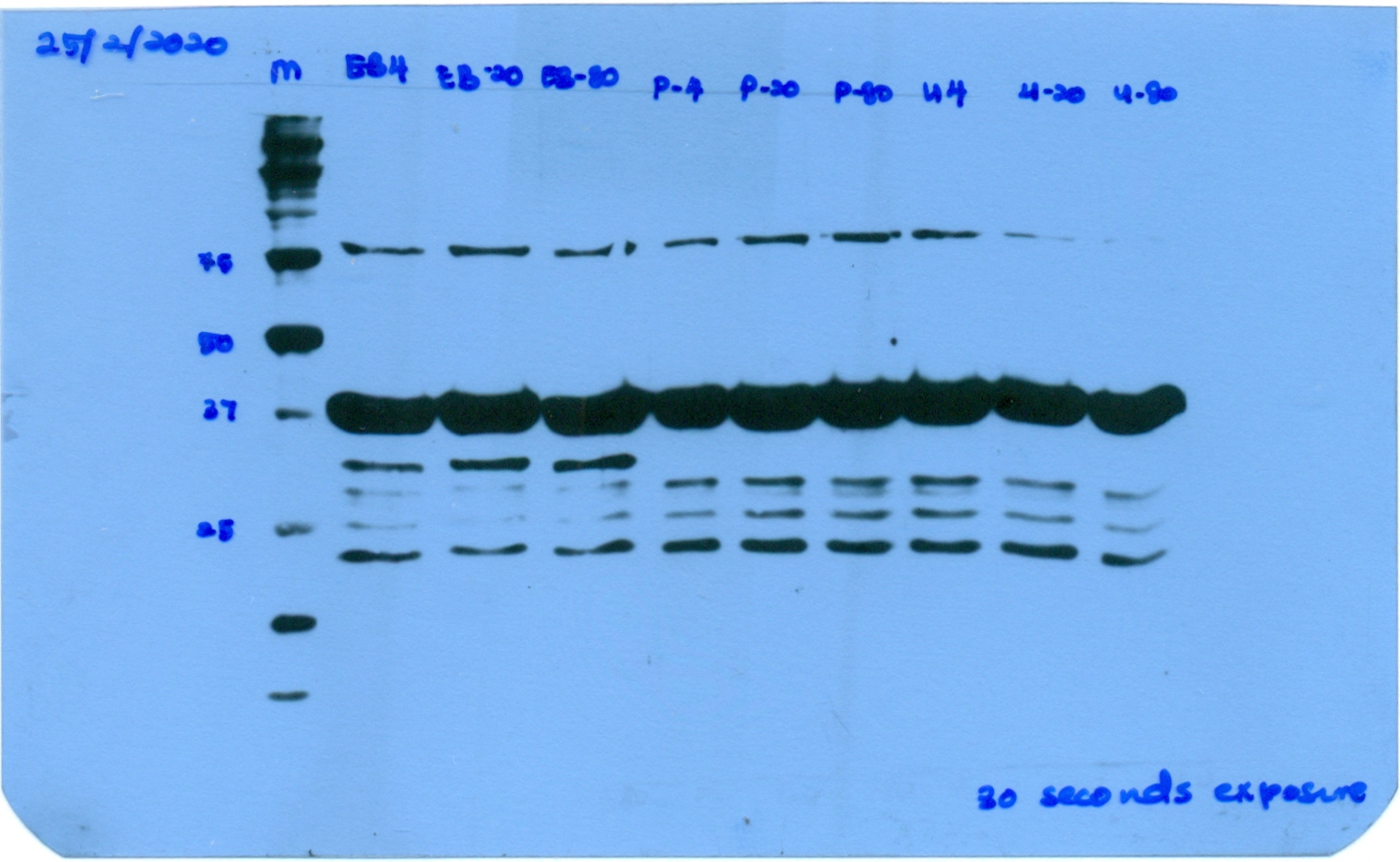


25kDa

30kDa

37kDa

50kDa

20kDa

**Figure S1**. SDS PAGE and Western blot analysis of rNIE antigen. a) SDS PAGE of rNIE. b) Western Blot analysis of rNIE. c) Original SDS PAGE image. d) Original Western Blot image.


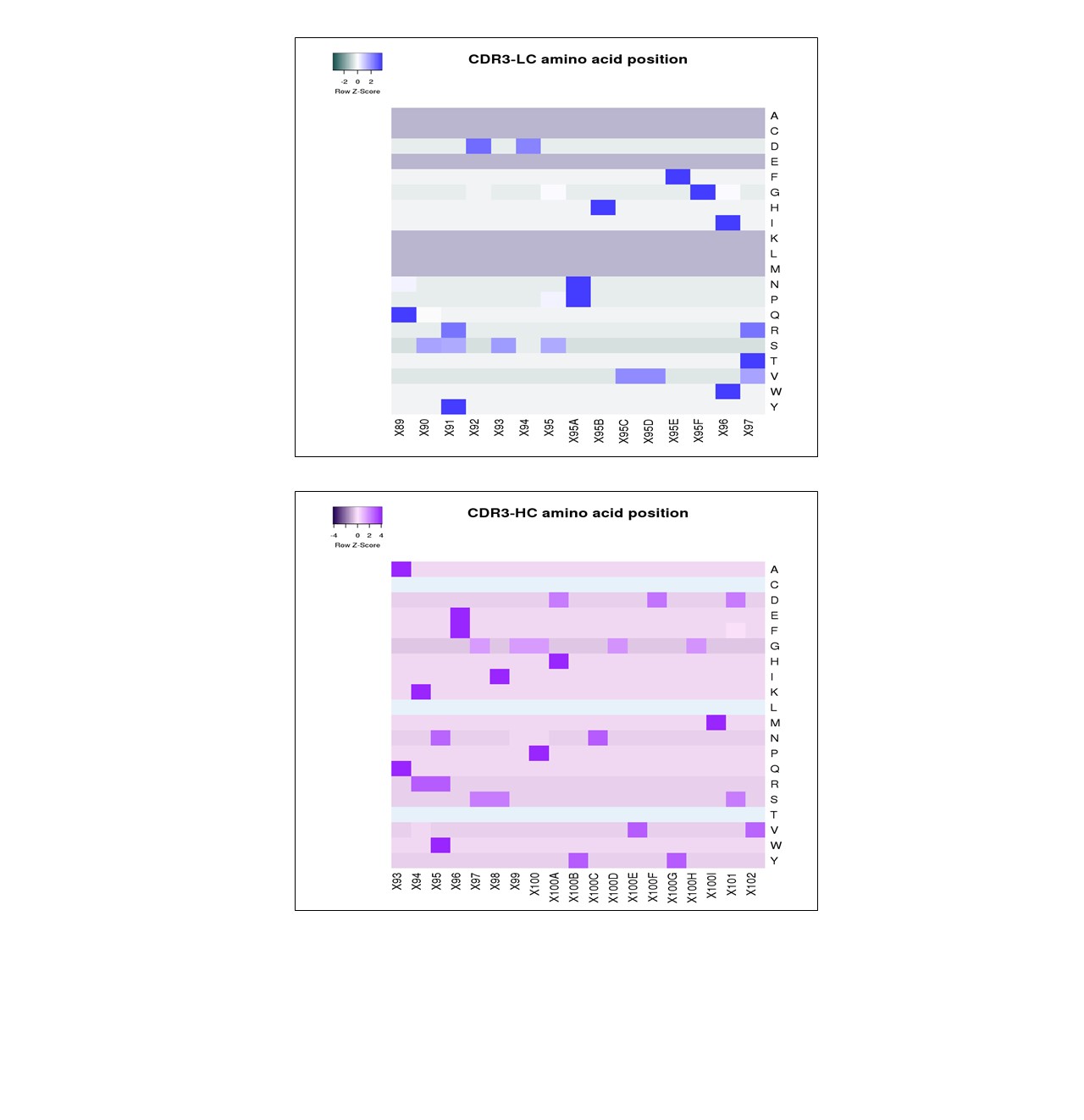


**Figure S2**. Heat map of rNIE specific monoclonal antibodies amino acid position at CDR3 position for heavy and light chains.


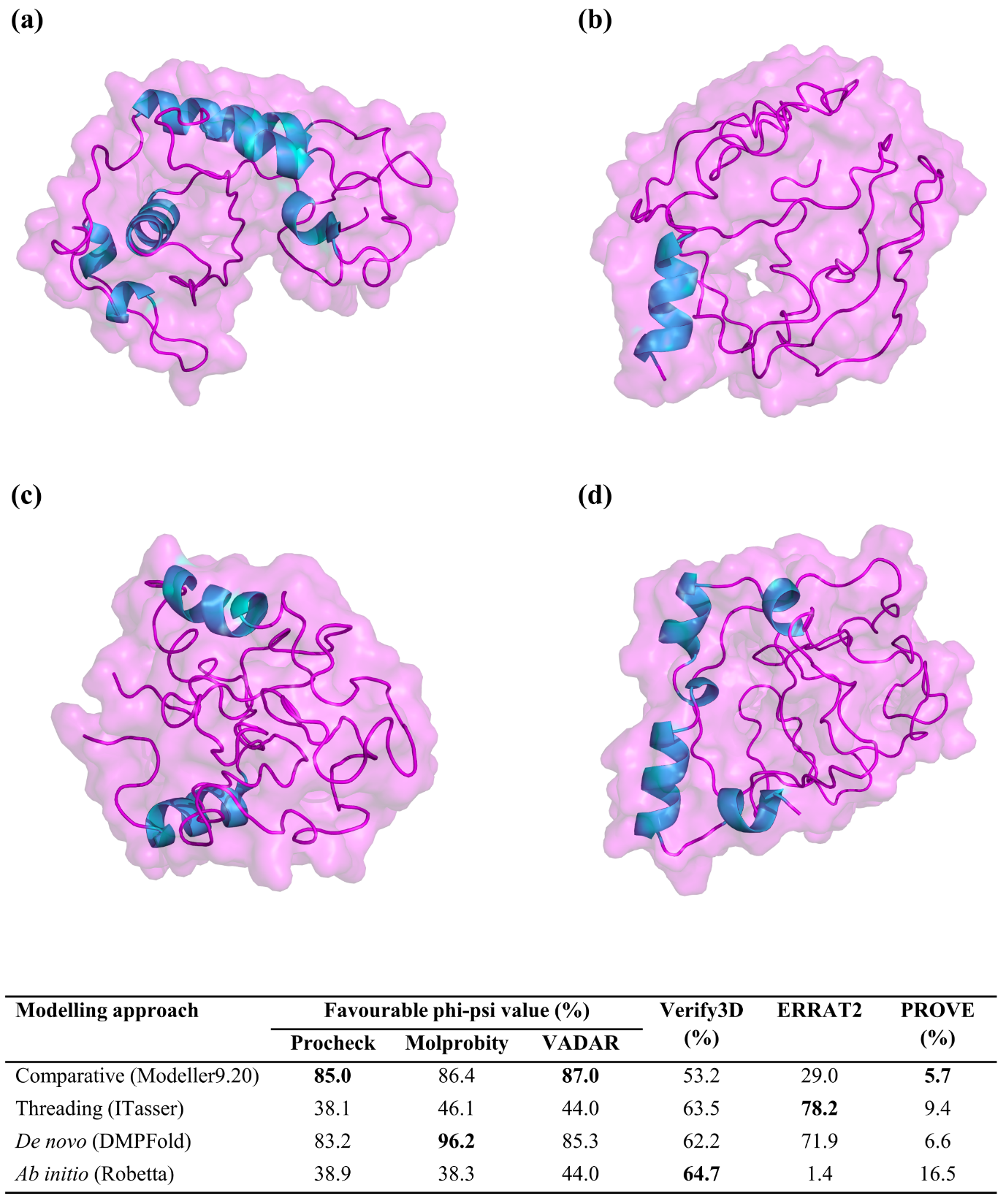


**Figure S3**. Three-dimension structure of rNIE protein modelled via different approaches. (a) MODELLERv9.20. (b) Itasser. (c) DMPFold. (d) Robetta.


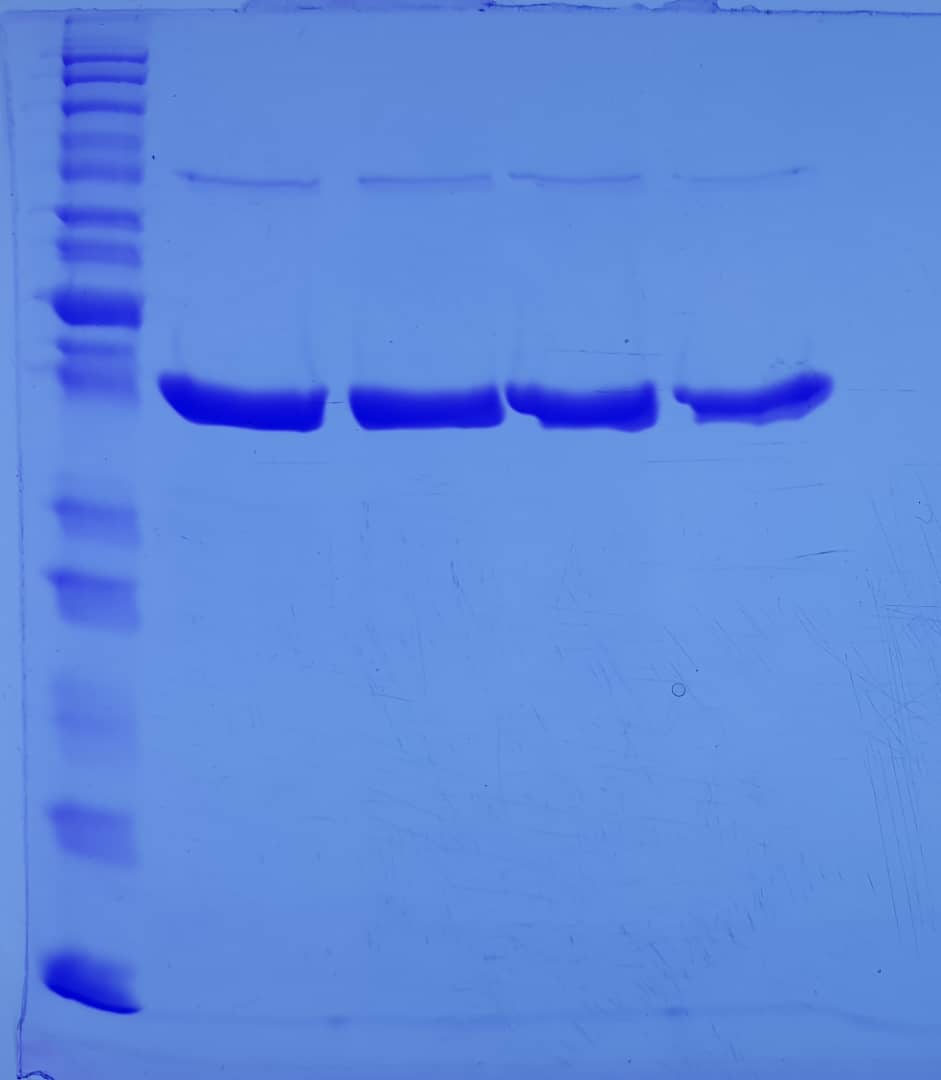


**M Ab5 Ab6 Ab14 Ab23**

50 kDa

40 kDa

30 kDa

25 kDa

20 kDa

15 kDa

10 kDa

**Figure S4**. SDS PAGE analysis of recombinant monoclonal antibody proteins.


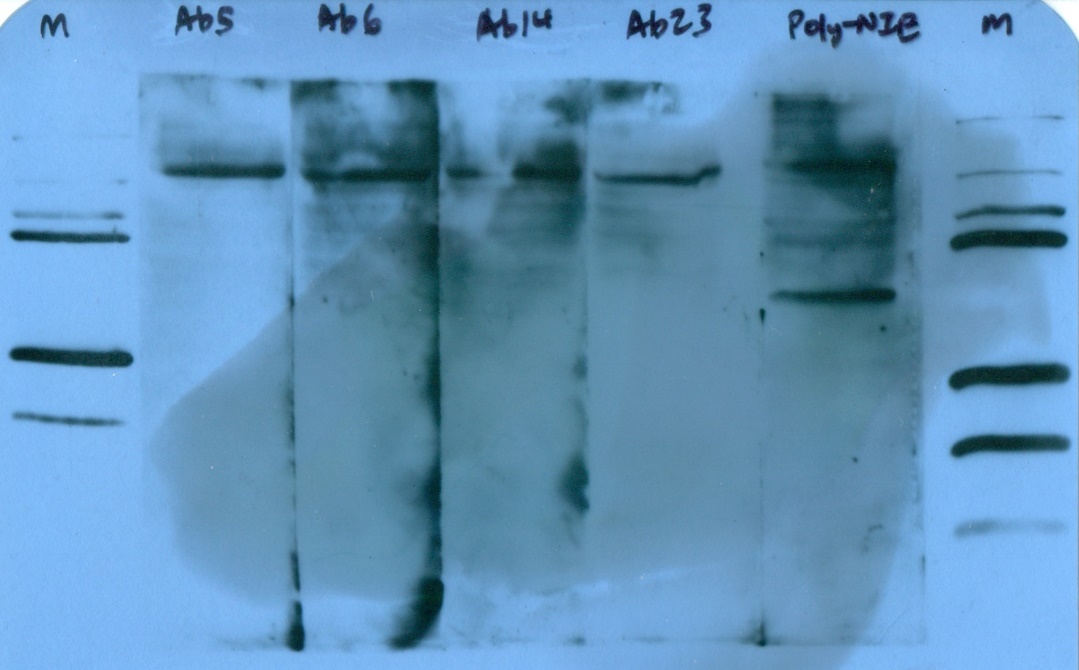

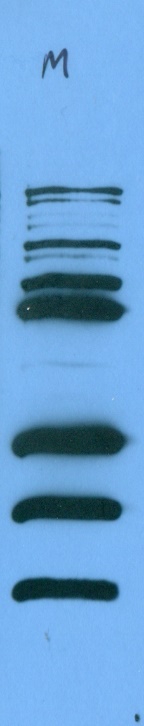


25kDa

30kDa

40kDa

50kDa

60kDa

M

Ab5

Ab6

Ab14

Ab23

Poly-anti-NIE

**a**

**b**


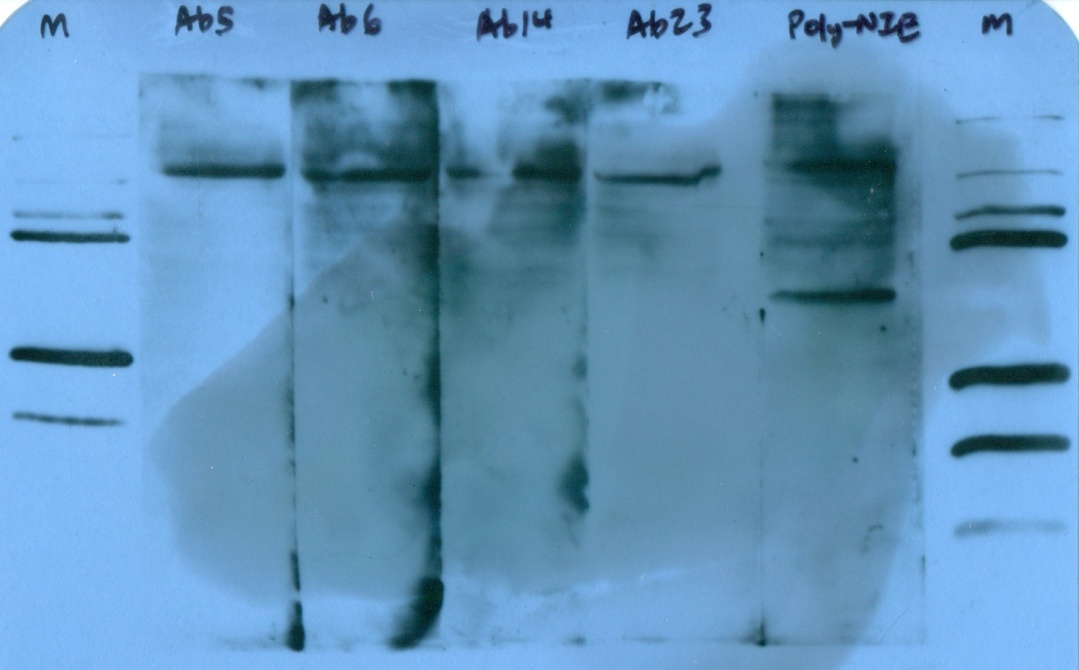


40kDa

50kDa

30kDa

25kDa

60kDa

**Figure S5**. Native antigen Western blot analysis. a) Rearranged figure of the ladder position. b) Original image.

**Figure S6**. Native antigen ELISA analysis.

10 20 30 40 50

....|....|....|....|....|....|....|....|....|....|

**NIE rAg**  **----------------------------------NSARVENQDQKDQLEN**

**Bm10733**  **------------------------MELQFSEFHMKICSVHQGS--PGGPA**

**Bm5878**  **MLRILVLFFSLSSLLPSFIN-GLAGNYSTNDGNMTVFEVSNAVKTDETPV**

**VIO88635**  **--MLTQKLSELSVFSTSFVFAGLAGNYSTNDGNMTVFEVSNAVKTDETPV**

**XP_001902196**  **------------MKYGNCVGYGLAGNYSTNDGNMTVFEVSNAVKTDETPV**

**Bm4214**  **----------------------------------MICSVHQGSRSPGGPT**

**XP_001900742**  **-----------MSVENATLGAIIFSGATLLVCLLAIAAIHKDVTSIWSEL**

60 70 80 90 100

....|....|....|....|....|....|....|....|....|....|

**NIE rAg**  **Q-------------------------------------------------**

**Bm10733**  **G-------------------------------------------------**

**Bm5878**  **DNLTSVLNATEIESLCLRMHNNTAFMGIQPFTRLMLYSASECRRNCVDLY**

**VIO88635**  **DNLTSVLNATEIESLCLRMHNNTAFMGIQPFTRLMLYSASECRRNCVDLY**

**XP_001902196**  **DNLTSVLNATEIESLCLRMHNNTAFMGIQPFTRLMLYSASECRRNCVDLY**

**Bm4214**  **G-------------------------------------------------**

**XP_001900742**  **DSEIVSFKVKTDDLWKEMIG------------------------------**

110 120 130 140 150

....|....|....|....|....|....|....|....|....|....|

**NIE rAg**  **-----------------------------DQKDQLENQDQK---------**

**Bm10733**  **--------------------------SLGSGSSRLPGGPGK---------**

**Bm5878**  **PKCVAVMFYYLHEREKNHICYLFSKNSIDEDVALVPEKPMKKLDMIRSLE**

**VIO88635**  **PKCVAVMFYYLHEREKNHICYLFSKNSIDEDVALVPEKPMKKLDMIRSLE**

**XP_001902196**  **PKCVAVMFYYLHEREKNHICYLFSKNSIDEDVALVPEKPMKKLDMIRSLE**

**Bm4214**  **--------------------------SLGSGSSRLPGGPGK---------**

**XP_001900742**  **-------------------------LGAGTPSNRLRRQTSYN---IYDKG**

160 170 180 190 200

....|....|....|....|....|....|....|....|....|....|

**NIE rAg**  **---------------NQLKNQSENQDQKNQLKNQSENQDQKKPIK--KPI**

**Bm10733**  **-----------NEIYQATISPGGAGGPKLLDGILSGPSEPLGPQ---SPC**

**Bm5878**  **IVADCHQFDPFPPLFTDFTTSTDGVSKKKRDVGYDRPIETTGPWSAWSEC**

**VIO88635**  **IVADCHQFDPFPPLFTDFTTSTDGVSKKKRDVGYDRPIETTGPWSAWSEC**

**XP_001902196**  **IVADCHQFDPFPPLFTDFTTSTDGVSKKKRDVGYDRPIETTGPWSAWSEC**

**Bm4214**  **-----------NEICQATISPGDAGGPKLLDGIISGPGEPLGPR---SPC**

**XP_001900742**  **KKNDGNAYDSSNGSSSKYDSLSSGNSGSNYGSSASASSYDSGNSDGGYGY**

210 220 230 240 250

....|....|....|....|....|....|....|....|....|....|

**NIE rAg**  **KKPGP-------KPIRPIVK-PKPKTTTQAPEEPEGPEEPEG--------**

**Bm10733**  **DPGGP-------KLLDGIISGPGEPLGPRGPCDPGGPKLLDG--------**

**Bm5878**  **STRSG-------RQVRSQLCEYGRNIQRRRCSSSASHHALGYGVKITSYA**

**VIO88635**  **STRSG-------RQVRSQLCEYGRNIQRRRCSSSASHHALGYGVKITSYA**

**XP_001902196**  **STRSG-------RQVRSQLCEYGRNIQRRRCSSSASHHALGYGVKITSYA**

**Bm4214**  **DLGGP-------KLLDGILSGPGEPLDPRGPCDPGGPKLLDG--------**

**XP_001900742**  **GAGGTNYVCSLENTCPPGSPGPKGEKGVDGENGIPGKDGIDGIDADDIQQ**

260 270 280 290 300

....|....|....|....|....|....|....|....|....|....|

**NIE rAg**  **---------PEEPEGPEGPEEPEGP-------------------------**

**Bm10733**  **-----IISGPDEPLDPSGPCDPGRP-LIP----------LPR-LYHHQFL**

**Bm5878**  **PSIDISISYPPYPYQHPDVSSDEYKRIMSAHSKQMAQSCCYWQDYFRQKT**

**VIO88635**  **PSIDISISYPPYPYQHPDVSSDEYKRIMSAHSKQMAQSCCYWQDYFRQKT**

**XP_001902196**  **PSIDISISYPPYPYQHPDVSSDEYKRIMSAHSKQMAQSCCYWQDYFRQKT**

**Bm4214**  **-----ILSGPGEPLDPRSPCDPGGPKLLDGILSGPGEPLDPRGPCDPGGP**

**XP_001900742**  **ESPSGCFNCPEGPPGPPGP--LGRPGIRGQRGPKGAPGFPGRDGNPGPPG**

310 320 330 340 350

....|....|....|....|....|....|....|....|....|....|

**NIE rAg**  **---------------------------------------AGPEEPEGPAG**

**Bm10733**  **NLMNRLCPGEMQKLKMKNEMEAIS--------KMKN--KVGVEELKEDGA**

**Bm5878**  **NLANGHKMQIQQQLGVRDGCPTTCSTSTQSKLQPLPYSQPQPQAPVYSQP**

**VIO88635**  **NLANGHKMQIQQQLGVRDGCPTTCSTSTQSKLQPLPYSQPQPQAPVYSQP**

**XP_001902196**  **NLANGHKMQIQQQLGVRDGCPTTCSTSTQSKLQPLPYSQPQPQAPVYSQP**

**Bm4214**  **KLLDGILSGPGEPLDPRSPCDPGGP-------KLLDGILSGPGEPLDPRG**

**XP_001900742**  **DIGPPGPPGLDGKPGEPGEKGADAEKVVGRKGNRGPPGDQGPEGPPGDKG**

360 370 380 390 400

....|....|....|....|....|....|....|....|....|....|

**NIE rAg**  **----PEEPE-------------------------------GPEEPEGPAG**

**Bm10733**  **----KFRLGRFGAQFFAPVTN-------------------EPSD-ISSAG**

**Bm5878**  **----QPQAPVYGPQPQLPVYGPQPQPSVYGLQPQAPVYGPQPQAPVYGPQ**

**VIO88635**  **----QPQAPVYGPQPQLPVYGPQPQPSVYGLQPQAPVYGPQPQAPVYGPQ**

**XP_001902196**  **----QPQAPVYGPQPQLPVYGPQPQPSVYGLQPQAPVYGPQPQAPVYGPQ**

**Bm4214**  **----PCDPG--GPKLLDGILS-------------------GPGEPLDPRG**

**XP_001900742**  **KDASPGEPGPEGPAGQPGFQGPQG--------------SDGDEGPEGPSG**

410 420 430 440 450

....|....|....|....|....|....|....|....|....|....|

**NIE rAg**  **PEEPR----------DDDDGVDEEDERD----------------------**

**Bm10733**  **DDDDD----------DGDEEVESEDSEDEK--------------------**

**Bm5878**  **PQAPVYGPQPQAPVYGPQPQVPVYGPQPQLPADVQAPISVWSSWSEWSFC**

**VIO88635**  **PQAPVYGPQPQAPVYGPQPQVPVYGPQPQLPADVQAPISVWSSWSEWSFC**

**XP_001902196**  **PQAPVYGPQPQAPVYGPQPQVPVYGPQPQLPADVQAPISVWSSWSEWSFC**

**Bm4214**  **PCDPGRSLIPLPRLYHHQAHLPHQKSPGASESPILPPLPGLPGMP-----**

**XP_001900742**  **NPGKDAEYCPCPARGHNAAHRLHGSKGNDDNESKTDDVQFKSDEESGQFG**

460 470 480 490 500

....|....|....|....|....|....|....|....|....|....|

**NIE rAg**  **--------------------------------------------------**

**Bm10733**  **--------------------------------------------------**

**Bm5878**  **SATCGIGMIQRYRICNTGQCKGENVEWRTCHQTVPCVASWADWTSWSSCS**

**VIO88635**  **SATCGIGMIQRYRICNTGQCKGENVEWRTCHQTVPCVASWADWTSWSSCS**

**XP_001902196**  **SATCGIGMIQRYRICNTGQCKGENVEWRTCHQTVPCVASWADWTSWSSCS**

**Bm4214**  **--------------------------------------------------**

**XP_001900742**  **KTDFENPNDSDKDYKSRRTLL-----------------------------**

510 520 530 540 550

....|....|....|....|....|....|....|....|....|....|

**NIE rAg**  **--------------------------------------------------**

**Bm10733**  **--------------------------------------------------**

**Bm5878**  **ATCGMGEKTRSRYCYLGANYCTGSDHEVTQCETAPCPGWGQWESWSYCSV**

**VIO88635**  **ATCGMGEKTRSRYCYLGANYCTGSDHEVTQCETAPCPGWGQWESWSYCSV**

**XP_001902196**  **ATCGMGEKTRSRYCYLGANYCTGSDHEVTQCETAPCPGWGQWESWSYCSV**

**Bm4214**  **--------------------------------------------------**

**XP_001900742**  **--------------------------------------------------**

560 570 580 590 600

....|....|....|....|....|....|....|....|....|....|

**NIE rAg**  **--------------------------------------------------**

**Bm10733**  **--------------------------------------------------**

**Bm5878**  **TCGTGIKRRTRICNGDKCIGDTYQESHCYQEQCEGWSEWQEWSACSVSCG**

**VIO88635**  **TCGTGIKRRTRICNGDKCIGDTYQESHCYQEQCEGWSEWQEWSACSVSCG**

**XP_001902196**  **TCGTGIKRRTRICNGDKCIGDTYQESHCYQEQCEGWSEWQEWSACSVSCG**

**Bm4214**  **--------------------------------------------------**

**XP_001900742**  **--------------------------------------------------**

610 620 630 640 650

....|....|....|....|....|....|....|....|....|....|

**NIE rAg**  **--------------------------------------------------**

**Bm10733**  **--------------------------------------------------**

**Bm5878**  **EGVIIRERVCMGRNCIGESSEQNICVEEACSTWQEWGEWSTCSAKCNFGI**

**VIO88635**  **EGVIIRERVCMGRNCIGESSEQNICVEEACSTWQEWGEWSTCSAKCNFGI**

**XP_001902196**  **EGVIIRERVCMGRNCIGESSEQNICVEEACSTWQEWGEWSTCSAKCNFGI**

**Bm4214**  **--------------------------------------------------**

**XP_001900742**  **--------------------------------------------------**

660 670 680 690 700

....|....|....|....|....|....|....|....|....|....|

**NIE rAg**  **--------------------------------------------------**

**Bm10733**  **--------------------------------------------------**

**Bm5878**  **STRRRLCHGIFCPGKRMEVMPCHAGRCAMWSTWQEWSECSVTCDSGIQQR**

**VIO88635**  **STRRRLCHGIFCPGKRMEVMPCHAGRCAMWSTWQEWSECSVTCDSGIQQR**

**XP_001902196**  **STRRRLCHGIFCPGKRMEVMPCHAGRCAMWSTWQEWSECSVTCDSGIQQR**

**Bm4214**  **--------------------------------------------------**

**XP_001900742**  **--------------------------------------------------**

710 720 730 740 750

....|....|....|....|....|....|....|....|....|....|

**NIE rAg**  **--------------------------------------------------**

**Bm10733**  **--------------------------------------------------**

**Bm5878**  **YRNCIGDNCMGSTEDMKYCETGISCPQWTKWTAWSRCSHDCGIGERIRYR**

**VIO88635**  **YRNCIGDNCMGSTEDMKYCETGISCPQWTKWTAWSRCSHDCGIGERIRYR**

**XP_001902196**  **YRNCIGDNCMGSTEDMKYCETGISCPQWTKWTAWSRCSHDCGIGERIRYR**

**Bm4214**  **--------------------------------------------------**

**XP_001900742**  **--------------------------------------------------**

760 770 780 790 800

....|....|....|....|....|....|....|....|....|....|

**NIE rAg**  **--------------------------------------------------**

**Bm10733**  **--------------------------------------------------**

**Bm5878**  **ECSSAGEPTDSCEGQKSETAVCLERLCCEWTAWTSWTPCSHMCGTGRSSR**

**VIO88635**  **ECSSAGEPTDSCEGQKSETAVCLERLCCEWTAWTSWTPCSHMCGTGRSSR**

**XP_001902196**  **ECSSAGEPTDSCEGQKSETAVCLERLCCEWTAWTSWTPCSHMCGTGRSSR**

**Bm4214**  **--------------------------------------------------**

**XP_001900742**  **--------------------------------------------------**

810 820 830 840 850

....|....|....|....|....|....|....|....|....|....|

**NIE rAg**  **--------------------------------------------------**

**Bm10733**  **--------------------------------------------------**

**Bm5878**  **TKMCLRLGYEQDDSSCTGQCYGNSREDKTCSEQISCATPTYPPICVRVTG**

**VIO88635**  **TKMCLRLGYEQDDSSCTGQCYGNSREDKTCSEQISCATPTYPPICVRVTG**

**XP_001902196**  **TKMCLRLGYEQDDSSCTGQCYGNSREDKTCSEQISCATPTYPPICVRVTG**

**Bm4214**  **--------------------------------------------------**

**XP_001900742**  **--------------------------------------------------**

860 870 880 890 900

....|....|....|....|....|....|....|....|....|....|

**NIE rAg**  **--------------------------------------------------**

**Bm10733**  **--------------------------------------------------**

**Bm5878**  **YTPGLPSTAVAPSTDGSCPPGYVLAPGVSGYIPTSITPGYTPTSITPGYI**

**VIO88635**  **YTPGLPSTAVAPSTDGSCPPGYVLAPGVSGYIPTSITPGYTPTSITPGYI**

**XP_001902196**  **YTPGLPSTAVAPSTDGSCPPGYVLAPGVSGYIPTSITPGYTPTSITPGYI**

**Bm4214**  **--------------------------------------------------**

**XP_001900742**  **--------------------------------------------------**

910 920 930 940 950

....|....|....|....|....|....|....|....|....|....|

**NIE rAg**  **--------------------------------------------------**

**Bm10733**  **--------------------------------------------------**

**Bm5878**  **PASITSGYTPGISASGYVSAPETPDYTVVPTAAGYVPSPSVPGYFPPPST**

**VIO88635**  **PASITSGYTPGISASGYVSAPETPDYTVVPTAAGYVPSPSVPGYFPPPST**

**XP_001902196**  **PASITSGYTPGISASGYVSAPETPDYTVVPTAAGYVPSPSVPGYFPPPST**

**Bm4214**  **--------------------------------------------------**

**XP_001900742**  **--------------------------------------------------**

960 970 980 990 1000

....|....|....|....|....|....|....|....|....|....|

**NIE rAg**  **--------------------------------------------------**

**Bm10733**  **--------------------------------------------------**

**Bm5878**  **SGYPAAPGVSGYVFVPGTPDYVVASYAPGYVTLPSAPGYVTLPSAPGYVL**

**VIO88635**  **SGYPAAPGVSGYVFVPGTPDYVVASYAPGYVTLPSAPGYVTLPSAPGYVL**

**XP_001902196**  **SGYPAAPGVSGYVFVPGTPDYVVASYAPGYVTLPSAPGYVTLPSAPGYVL**

**Bm4214**  **--------------------------------------------------**

**XP_001900742**  **--------------------------------------------------**

1010 1020 1030 1040 1050

....|....|....|....|....|....|....|....|....|....|

**NIE rAg**  **--------------------------------------------------**

**Bm10733**  **--------------------------------------------------**

**Bm5878**  **PRSAPGYFLTPSGYAMPAGIFIEVRCHWAEWYQWSACEKTCYHHVKRRTR**

**VIO88635**  **PRSAPGYFLTPSGYAMPAGIFIEVRCHWAEWYQWSACEKTCYHHVKRRTR**

**XP_001902196**  **PRSAPGYFLTPSGYAMPAGIFIEVRCHWAEWYQWSACEKTCYHHVKRRTR**

**Bm4214**  **--------------------------------------------------**

**XP_001900742**  **--------------------------------------------------**

1060 1070

....|....|....|....|....|...

**NIE rAg**  **----------------------------**

**Bm10733**  **----------------------------**

**Bm5878**  **HCIGEDECMCSGDAQEEINCLLPPECRI**

**VIO88635**  **HCIGEDECMCSGDAQEEINCLLPPECRI**

**XP_001902196**  **HCIGEDECMCSGDAQEEINCLLPPECRI**

**Bm4214**  **----------------------------**

**XP_001900742**  **----------------------------**

**Figure S7**. ClustalW alignment of rNIE antigen against *B. malayi* proteins.


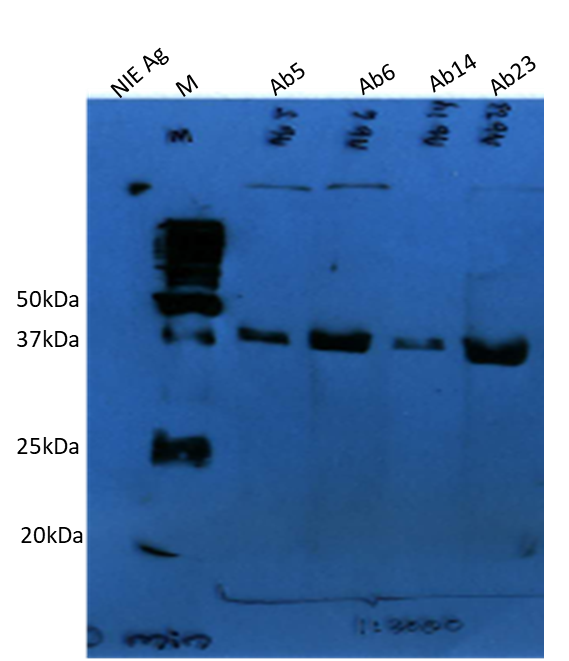


a


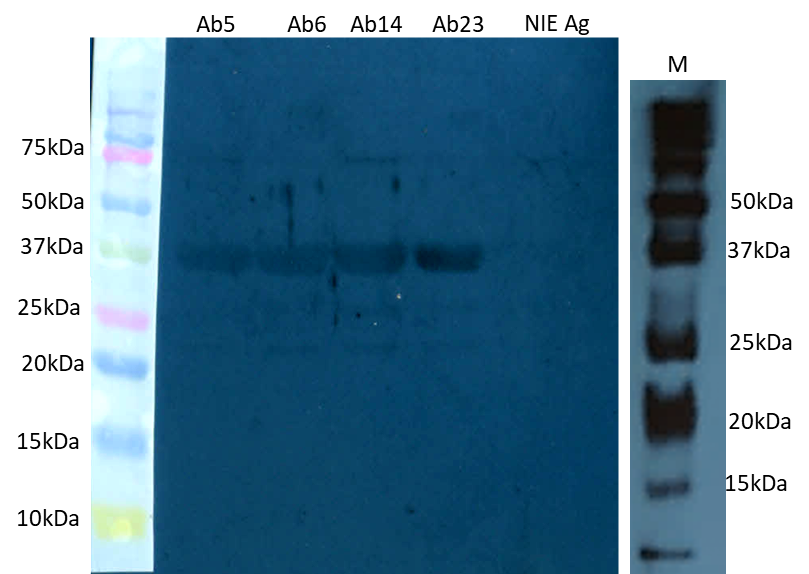


b

**Figure S8**. Original image of the verification and binding analysis of rNIE specific recombinant monoclonal antibody proteins of Figure 7 in text. (a) Original Western blot analysis of recombinant monoclonal antibody proteins image. (b) Original antigen-antibody Western blot analysis image.

**Table S1**. The secondary structure prediction (by PsiPred, JPred4 and Spider3) of rNIE and the secondary structure calculation (by PyMOL) of the rNIE models build by comparative modelling approach (MODELLER9.20).

Sequence NSARVENQDQ KDQLENQDQK DQLENQDQKN QLKNQSENQD QKNQLKNQSE NQDQKKPIKK

PSIPRED --HHHHHHHH HHHHHHHHHH HHHHHHHHH- ---------- ---------- ----------

JPred4 --HHHHHHHH HHHHHHHHHH HHH------- ---------- ---------- ----------

Spider3 ---------H HHHHHHHHHH HHHHHHHHH- H--------- ---------- ----------

Consesus --HHHHHHHH HHHHHHHHHH HHHHHHHHH- ---------- ---------- ----------

Comparative -HHHH----- -----HHHH- ---------- -------HHH HHHHHHHHHH HHH-------

Threading -HHHHHHHHH H--------- ---------- ---------- ---------- ----------

*De novo* -------HHH HHHHHHHH-- ---------- --------HH HHHHHHHH-- ----------

*Ab initio* -HHHHHH--- HHHHHHHHHH -HHH-HHHHH HH—-HHHHH- ---------- ----------

120

Sequence PIKKPGPKPI RPIVKPKPKT TTQAPEEPEG PEEPEGPEEP EGPEGPEEPE GPAGPEEPEG

PSIPRED ---------- ---------- ---------- ---------- ---------- ----------

JPred4 --------- ---------- ---------- ---------- ---------- ----------

Spider3 ---------- ---------- ---------- ---------- ---------- ----------

Consesus ---------- ---------- ---------- ---------- ---------- ----------

Comparative ---------- -----HHHHH HHHHHHH--- ---------- ---------- -------HHH

Threading ---------- ---------- ---------- ---------- ---------- ----------

*De novo*  ---------- ---------- ---------- ---------- ---------- ----------

*Ab initio*  ---------- ---------- ---------- ---------- ---------- ----------

156

Sequence PAGPEEPEGP EEPEGPAGPE EPRDDDDGVD EEDERD

PSIPRED ---------- ---------- ---------- ------

JPred4 ---------- ---------- ---------- ------

Spider3 ---------- ---------- ---------- ------

Consesus ---------- ---------- ---------- ------

Comparative HHHHHH---- ---------- HHHHH----- ------

Threading ---------- ---------- ---------- ------

*De novo* ---------- ---------- ---------- ------

*Ab initio*  ---------- ---------- ---------- ------

H: Helix; -: Coil/Turn

**Table S2.** The stereochemistry evaluation for the structure of rNIE antigen constructed by four different approaches. The best scores for each evaluation were highlighted in bold.

| **Modelling approach** | **Favourable phi-psi value (%)** | | | **Verify3D (%)** | **ERRAT2**  **(quality factor)** | **PROVE (%)** |
| --- | --- | --- | --- | --- | --- | --- |
|  | **Procheck** | **Molprobity** | **VADAR** |  |  |  |
| Comparative (Modeller) | **85.0** | 86.4 | **87.0** | 53.2 | 29.0 | **5.7** |
| Threading (ITasser) | 38.1 | 46.1 | 44.0 | 63.5 | **78.2** | 9.4 |
| Threading (DMPFold) | 83.2 | **96.2** | 85.3 | 62.2 | 71.9 | 6.6 |
| Ab initio (Robetta) | 38.9 | 38.3 | 44.0 | **64.7** | 1.4 | 16.5 |

**Table S3**. The intermolecular interactions observed between antibody clones and rNIE.

| **Clone** | **Chain** | **CDR** | **Clone residue** | **NIE residue** | **Distance (Å)** | **Interaction** |
| --- | --- | --- | --- | --- | --- | --- |
| Ab5 | Heavy | 2 | Ile54 CD1 | Gln28 HE22 | 3.1 | Hydrophobic |
|  |  |  | Val59 CG1 | Gln28.HE22 | 3.2 | Hydrophobic |
|  |  | 3 | Cys105 CB | Asp21 OD2 | 3.6 | Hydrophobic |
|  |  |  | Asn106 H | Asp21 OD2 | 2.2 | Hbond |
|  |  |  | Asn106 HD22 | Arg4 NH2 | 2.7 | Hydrophobic |
|  |  |  | Arg107 H | Asp21 OD2 | 2.1 | Hbond |
|  |  |  | Arg107 NH1 | Asp18 OD1 | 2.3 | Hydrophilic |
|  |  |  | Arg107 HH21 | Asn7 OD1 | 1.8 | Hbond |
|  |  |  | Arg107 HH22 | Gln17 O | 1.9 | Hbond |
|  |  |  | Tyr111 HH | Asp21 OD1 | 2.1 | Hbond |
|  |  |  | Tyr111 OH | Lys20 HZ1 | 1.7 | Hbond |
|  |  |  | Asn113 HD21 | Asp27 OD1 | 2.1 | Hydrophobic |
|  |  |  | Tyr114 H | Asp27 OD1 | 2.2 | Hydrophobic |
|  |  |  | His115 CE1 | Gln22 CG | 3.8 | Hydrophobic |
|  |  |  | His115 H | Asp27 OD1 | 1.9 | Hbond |
|  |  |  | His115 ND1 | Glu24 OE2 | 2.2 | Hydrophilic |
|  |  |  | Tyr116 HH | Asp21 OD1 | 1.9 | Hbond |
|  |  |  | Tyr116 HH | Gln22 CB | 3.3 | Hydrophobic |
|  |  |  | Tyr116 OH | Lys20 HZ2 | 2.5 | Hydrophobic |
|  | Light | 1 | Ser188 CB | Asn34 OD1 | 3.2 | Hydrophobic |
|  |  |  | Leu190 CD1 | Gln22 NE2 | 3.6 | Hydrophobic |
|  |  |  | Leu190 H | Glu24 OE1 | 2.0 | Hbond |
|  |  | 2 | Ser208 OG | Gln22 HE22 | 1.9 | Hbond |
|  |  | 3 | Trp249 CZ2 | Asn30 ND2 | 3.7 | Hydrophobic |
|  |  |  | Trp249 HE1 | Asp27 CB | 3.2 | Hydrophobic |
|  |  |  | Ser252 HG | Asn30 OD1 | 1.9 | Hbond |
| **Clone** | **Chain** | **CDR** | **Clone residue** | **NIE residue** | **Distance (Å)** | **Interaction** |
| Ab6 | Heavy | 2 | Trp55B CE2 | Lys20 HZ3 | 2.4 | Hydrophobic |
|  |  |  | Trp55 HE1 | Gln19 O | 2.7 | Hydrophobic |
|  |  |  | Asp56 OD1 | Lys20 NZ | 1.8 | Hydrophobic |
|  |  |  | Asp56 OD2 | Gln26 HE21 | 2.1 | Hbond |
|  |  |  | Ser57 HG | Asp27 OD1 | 1.9 | Hbond |
|  |  |  | Ser57 HG | Gln28 HE21 | 2.3 | Hydrophobic |
|  |  |  | Ser57 O | Gln26 CA | 3.1 | Hydrophobic |
|  |  |  | Asn58 CB | Glu24 O | 3.3 | Hydrophobic |
|  |  |  | Asn58 HD22 | Lys20 HZ2 | 2.4 | Hydrophobic |
|  |  |  | Asn58 HD22 | Gln26 NE2 | 2.9 | Hydrophobic |
|  |  |  | Asn58 OD1 | Asn25 C | 3.1 | Hydrophobic |
|  |  |  | Ser59 CA | Gln22 CB | 3.3 | Hydrophobic |
|  |  |  | Ser59 HG | Asp21 OD2 | 2.0 | Hbond |
|  |  |  | Arg60 OE1 | Glu24 OE1 | 1.9 | Hydrophilic |
|  |  |  | Arg60 HH21 | Asn25 O | 1.8 | Hbond |
|  |  |  | Arg60 NE | Asp27 OD2 | 1.9 | Hydrophilic |
|  |  | 3 | Ile104 CD1 | Gln19 CG | 3.8 | Hydrophobic |
|  |  |  | Gly105 CA | Asp21 OD1 | 3.4 | Hydrophobic |
|  |  |  | Gly106 H | Asp21 OD1 | 2.1 | Hydrophobic |
|  |  |  | Asp107 H | Asp21 OD2 | 2.1 | Hbond |
|  |  |  | Asp107 OD1 | Arg4 NH1 | 1.8 | Hydrophilic |
|  |  |  | Tyr108 CE2 | Arg4 HH12 | 2.8 | Hydrophobic |
|  |  |  | Tyr108 HH | Gln17 O | 1.9 | Hbond |
|  |  |  | Tyr108 HH | Asp18 CA | 2.9 | Hydrophobic |
|  |  |  | Tyr108 OH | Asn7 HD22 | 2.2 | Hbond |
|  |  |  | Asn109 HD22 | Asp18 OD2 | 2.0 | Hbond |
| **Clone** | **Chain** | **CDR** | **Clone residue** | **NIE residue** | **Distance (Å)** | **Interactions** |
| Ab14 | Heavy | 1 | Thr21 HG1 | Asp18 OD1 | 2.0 | Hbond |
|  |  |  | Ser22 HG | Asp18 OD1 | 1.9 | Hbond |
|  |  |  | Ser22 O | Asp21 CB | 3.0 | Hydrophobic |
|  |  |  | Ser22 OG | Arg4 HH21 | 1.9 | Hbond |
|  |  |  | Tyr23 CA | Asp21 OD2 | 3.3 | Hydrophobic |
|  |  |  | Trp24 CZ2 | Lys20 HZ2 | 2.4 | Hydrophobic |
|  |  |  | Trp24 H | Asp21 OD2 | 2 | Hydrophobic |
|  |  |  | Trp24 HE1 | Gln19 O | 2.8 | Hydrophobic |
|  |  | 2 | Glu43 OE1 | Lys20 NZ | 1.8 | Hydrophilic |
|  |  |  | Glu43 OE2 | Gln19 HE21 | 2.0 | Hbond |
|  |  |  | Asp46 OD1 | Gln19 HE21 | 2.7 | Hydrophobic |
|  |  | 3 | Arg90 C | Asp21 OD2 | 3.3 | Hydrophobic |
|  |  |  | Arg90 HE | Gln22 HE21 | 2.2 | Hydrophobic |
|  |  |  | Arg90 NH1 | Asp27 OD2 | 1.8 | Hydrophilic |
|  |  |  | Arg90 NH2 | Glu24 OE2 | 1.8 | Hydrophilic |
|  |  |  | Gly91 H | Gln22 NE2 | 2.5 | Hydrophobic |
|  |  |  | Gly91 N | Asp21 OD2 | 3.1 | Hydrophobic |
|  |  |  | Gly92 O | Gln22 HE22 | 1.9 | Hbond |
|  |  |  | Gly93 N | Gln22 HE22 | 3.0 | Hydrophobic |
|  | Light | 1 | Ser163 O | Asn30 HD22 | 2.0 | Hydrophobic |
|  |  |  | Ser165 CA | Gln31 O | 3.1 | Hydrophobic |
|  |  |  | Ser165 HG | Asn25 OD1 | 1.8 | Hbond |
|  |  |  | Ser165 O | Asn34 HD22 | 2.3 | Hbond |
|  |  |  | Ser166 HG | Asn34 OD1 | 1.8 | Hbond |
|  |  |  | Asn167 HD21 | Glu24 OE2 | 2.0 | Hbond |
|  |  | 3 | Tyr226 C | Asp27 OD1 | 3.4 | Hydrophobic |
|  |  |  | Tyr226 CB | Glu24 OE2 | 3.1 | Hydrophobic |
|  |  |  | Gly227 CA | Asp27 CA | 3.7 | Hydrophobic |
|  |  |  | Ser228 C | Asp27 O | 2.9 | Hydrophobic |
|  |  |  | Ser228 CB | Asn30 HD22 | 3.1 | Hydrophobic |
|  |  |  | Ser229 H | Asn30 OD1 | 2.2 | Hbond |
|  |  |  | Ser229 HG | Gln28 OE1 | 1.9 | Hbond |
|  |  |  | Ser229 N | Asp27 O | 3.0 | Hydrophobic |
|  |  |  | Pro231 CB | Asp27 CB | 4.1 | Hydrophobic |
|  |  |  | Pro231 CG | Gln28 NE2 | 3.6 | Hydrophobic |
|  |  |  | Ile232 CG1 | Asp27 OD1 | 3.7 | Hydrophobic |
| **Clone** | **Chain** | **CDR** | **Clone residue** | **NIE residue** | **Distance (Å)** | **Interactions** |
| Ab23 | Heavy | 2 | Asp56 OD2 | Lys20 NZ | 1.8 | Hydrophilic |
|  |  |  | Ser57 HG | Asp27 OD1 | 2.1 | Hydrophobic |
|  |  |  | Asn58 HD22 | Glu24 O | 1.9 | Hbond |
|  |  |  | Asn58 OD1 | Gln22 CG | 3.2 | Hydrophobic |
|  |  |  | Ser59 HG | Asp21 OD1 | 1.8 | Hbond |
|  |  |  | Ser59 OG | Gln22 CB | 3.2 | Hydrophobic |
|  |  | 3 | Ile104 CD1 | Leu14 CD2 | 3.9 | Hydrophobic |
|  |  |  | Ile104 CD1 | Gln19 HE21 | 3.2 | Hydrophobic |
|  |  |  | Ile104 CG2 | Asp18 OD2 | 3.7 | Hydrophobic |
|  |  |  | Gly105 CA | Gln19 O | 3.5 | Hydrophobic |
|  |  |  | Gly105 CA | Asp21 OD1 | 3.2 | Hydrophobic |
|  |  |  | Gly105 N | Asp18 O | 3.3 | Hydrophobic |
|  |  |  | Gly106 H | Asp21 OD1 | 2.1 | Hydrophobic |
|  |  |  | Asp107 H | Asp21 OD2 | 2.3 | Hbond |
|  |  |  | Asp107 OD1 | Arg4 NH1 | 2.0 | Hydrophilic |
|  |  |  | Tyr108 CB | Asp18 OD1 | 3.1 | Hydrophobic |
|  |  |  | Tyr108 CD2 | Asn7 ND2 | 3.3 | Hydrophobic |
|  |  |  | Asn109 H | Asp18 OD1 | 2.0 | Hbond |
|  |  |  | Gly110 H | Asp18 OD1 | 2.1 | Hbond |
|  | Light | 1 | Ser185 HG | Glu6 OE2 | 2.0 | Hbond |
|  |  |  | Tyr186 HH | Glu6 OE1 | 2.1 | Hydrophobic |
|  |  |  | Tyr186 OH | Val5 O | 2.9 | Hydrophobic |
|  |  |  | Tyr186 OH | Asn7 H | 2.5 | Hydrophobic |
|  |  |  | Tyr187 HH | Asp9 OD1 | 2.0 | Hbond |
|  |  | 2 | Lys206 NZ | Asp9 OD2 | 1.8 | Hydrophilic |
|  |  | 3 | Arg246 HH21 | Glu6 C | 2.8 | Hydrophobic |
|  |  |  | Arg246 HH21 | Asn7 OD1 | 1.8 | Hbond |
|  |  |  | Arg246 HH22 | Val5 O | 2.2 | Hbond |
|  |  |  | Arg246 NH1 | Arg4 NH1 | 3.5 | Hydrophobic |
|  |  |  | Ser249 O | Val5 CG1 | 3.3 | Hydrophobic |
|  |  |  | Gly250 O | Arg4 HH11 | 2.0 | Hbond |
|  |  |  | Asn251 OD1 | Arg4 NH2 | 3.3 | Hydrophobic |

**Table S4**. Physicochemical properties of NIE specific antibodies.

| **Clone** | **Isoelectric point (pI)** | **Charge**  **(pH 7)** | **Interface area**  **(Å^2^)** | **Binding free energy**  **(G_Bind_; kcal/mol)** |
| --- | --- | --- | --- | --- |
| Ab5 | 8.8 | +2.3 | 797.7 | -5.6 |
| Ab6 | 5.2 | -4.8 | 614.5 | -5.6 |
| Ab14 | 8.9 | +2.2 | 848.9 | -7.8 |
| Ab23 | 8.3 | +0.1 | 740.2 | -6.1 |
